# Species Diversity and Virulence Potential of the *Beauveria bassiana* Complex and *Beauveria scarabaeidicola* Complex

**DOI:** 10.3389/fmicb.2022.841604

**Published:** 2022-03-04

**Authors:** Yao Wang, Qi Fan, Dong Wang, Wei-Qiu Zou, De-Xiang Tang, Preeyanat Hongthong, Hong Yu

**Affiliations:** ^1^Yunnan Herbal Laboratory, School of Ecology and Environmental Science, Yunnan University, Kunming, China; ^2^The International Joint Research Center for Sustainable Utilization of Cordyceps Bioresources in China and Southeast Asia, Yunnan University, Kunming, China; ^3^Faculty of Agricultural Technology, Rajamangala University of Technology, Thanyaburi, Thailand

**Keywords:** Taxonomy, *Beauveria*, phylogenetic analyses, new species, biological control

## Abstract

*Beauveria* is a very important fungal resource. Some *Beauveria* species have great economic and ecological value. Through surveying *Beauveria* in China and Thailand over the past 4 years, 15 *Beauveria* spp. were collected and identified. Three new species—namely, *B. polyrhachicola*, *B. songmingensis*, and *B. subscarabaeidicola*—were described and illustrated based on morphological characteristics and molecular data. The phylogenetic positions of the 15 species were evaluated according to phylogenetic inferences based on six loci (nr*SSU*, nr*LSU*, *TEF*, *RPB1*, *RPB2*, and *Bloc*). Nine species of *Beauveria* in our study were isolated from adult scarab beetles (Coleoptera: Scarabaeidae). The pathogenicity of the isolates from the *B. bassiana* complex and *B. scarabaeidicola* complex was determined with three bioassays using *B. mori* and *T. molitor* larvae, in addition to *Protaetia brevitarsis* adults. The results indicated that the *B. bassiana* complex isolates had great potential in the biocontrol of the three insects; by contrast, the *B. scarabaeidicola* complex isolates showed obvious host specificity and low virulence.

## Introduction

*Beauveria* is a very important fungal resource, with some species having great economic and ecological value (Zimmermann, 2007; Rehner et al., 2011; Wang Y. et al., 2020). *Beauveria bassiana* (Bals.-Criv.) Vuill. and *B. brongniartii* (Sacc.) Petch are well-known environmentally safe alternatives to using chemical pesticides to control agricultural pests (Zimmermann, 2007; Rehner et al., 2011). *Beauveria pseudobassiana* S.A. Rehner and Humber has also been shown to have great potential in the biocontrol of numerous insect pests (Wang Y. et al., 2020). The entomopathogenic fungi *Beauveria* spp. are a class of environmentally friendly fungal pathogens that play an important role in controlling insect populations in nature (Luo et al., 2018; McKinnon et al., 2018). Some *Beauveria* species, as endophytes or soil and rhizosphere inhabitants, have been considered for potential use as biocontrol agents against plant pathogens by concerned practitioners, such as agriculturalists and plant pathologists. These species can produce an array of bioactive metabolites that limit the growth of some fungal plant pathogens and induce plant systemic resistance against the pathogenic bacterium (Ownley et al., 2010).

*Beauveria bassiana* is the most widely used fungus available commercially for controlling agricultural and forestry pests (Li et al., 2011). Products based on this species have been developed in many countries around the world (Goettel et al., 2005; Faria and Wraight, 2007; Li et al., 2011). However, a growing body of molecular evidence has demonstrated that *B. bassiana*, originally known as a generalist with a global distribution, encompasses cryptic lineages adapted to specific hosts or ecologies (Li et al., 2011; Rehner et al., 2011). Many initially identified *B. bassiana* isolates may belong to any of the species in the *B. bassiana* complex, such as *B. rudraprayagi* Y. Agrawal, Mual and Shenoy, *B. staphylinidicola* (Kobayasi and Shimizu) B. Shrestha, Kepler and Spatafora, and *B. peruviensis* D.E. Bustamante, M.S. Calderon, M. Oliva, and S. Leiva (Rehner et al., 2011; Agrawal et al., 2014; Kepler et al., 2017; Bustamante et al., 2019). Therefore, the abovementioned mycoinsecticide formulations of *B. bassiana* are not likely all based on *B. bassiana*.

*Beauveria scarabaeidicola* (Kobayasi) S.A. Rehner and Kepler is widely distributed in Oceania and Asia and named after its host adult beetle (Coleoptera: Scarabaeidae). It was originally described as *Cordyceps scarabaeicola* occurring in its sexual morph on an adult scarab beetle in New Guinea (Kobayasi and Shimizu, 1976). *Cordyceps scarabaeicola* has also been reported occasionally from many Asian countries, including China, Japan, and Korea (Shrestha et al., 2014). In an important phylogenetic study of *Beauveria*, a new entomopathogenic species, *B. sungii* S.A. Rehner and R.A. Humber, was described as a scarab-killing pathogen (hosts of all *B. sungii* isolates were identified as scarabs) (Rehner et al., 2011). Later, however, Shrestha et al. (2014) demonstrated that the telemorphic stage of *B. sungii* was *C. scarabaeicola* based on morphological and phylogenetic evidence. Because *C. scarabaeicola* was described earlier than *B. sungii*, Kepler et al. (2017) recommended *B. scarabaeicola* as the name of this species. Recently, Chen et al. (2019) proposed a new species, *B. yunnanensis*, a Chinese species parasitic on Lepidoptera pupa buried in soil that was a sister lineage to *B. scarabaeicola*.

During surveys of entomopathogenic fungi from different regions in Yunnan Province, China, and Chiang Rai Province, Thailand, over the past 4 years, approximately 15 *Beauveria* spp. were found and identified (Table 1). In this study, we aimed to: (1) reveal the hidden species diversity of the *B. bassiana* complex and *B. scarabaeidicola* complex based on phylogenetic analyses and morphological observation and (2) assess the biocontrol potential of species in the *B. bassiana* complex and *B. scarabaeidicola* complex through pathological tests on the lepidopteran *Bombyx mori* and the coleopteran *Tenebrio molitor* larvae as well as *Protaetia brevitarsis* adults.

**TABLE 1:** Specimen information and GenBank accession numbers for sequences used in this study.

Taxon	Voucher information	Host/substrate	GenBank accession number				References
			
			*TEF*	*RPB1*	*RPB2*	*Bloc*	
*Cordyceps cicadae*	RCEF HP090724-31	Hemiptera: Cicadidae	MF416496	MF416653	MF416447		Kepler et al., 2017
*Cordyceps tenuipes*	ARSEF 5135	Lepidopteran pupa	JF416020	JN049896	JF416000		Kepler et al., 2012
*Beauveria acridophila*	HUA 179219*^T^*	Orthoptera: Acrididae	JQ958613	JX003857	JX003841		Sanjuan et al., 2014
*Beauveria acridophila*	HUA 179220	Orthoptera: Acrididae	JQ958614	JX003852	JX003842		Sanjuan et al., 2014
*Beauveria amorpha*	ARSEF 2641*^T^*	Hymenoptera: Formicidae	AY531917	HQ880880	HQ880952	HQ880739	Rehner et al., 2011
*Beauveria araneola*	GZAC 150317*^T^*	Araneae	KT961699	KT961701		KT961698	Chen et al., 2017
*Beauveria asiatica*	ARSEF 4850*^T^*	Coleoptera: Cerambycidae	AY531937	HQ880859	HQ880931	HQ880718	Rehner et al., 2011
*Beauveria asiatica*	**YFCC 5600**	**Coleoptera: Cerambycidae**	MN576996	MN576886	MN576940	MW168177	Wang Y. B. et al., 2020; **This study**
*Beauveria australis*	ARSEF 4598*^T^*	Soil	HQ880995	HQ880861	HQ880933	HQ880720	Rehner et al., 2011
*Beauveria baoshanensis*	CCTCC AF 2018011*^T^*	Coleoptera: Chrysomelidae	MG642897	MG642854	MG642867		Chen et al., 2019
*Beauveria bassiana*	ARSEF 1564*^T^*	Lepidoptera: Arctiidae	HQ880974	HQ880833	HQ880905	HQ880692	Rehner et al., 2011
*Beauveria bassiana*	ARSEF 7518	Hymenoptera: Pamphiliidae	HQ880975	HQ880834	HQ880906	HQ880693	Rehner et al., 2011
*Beauveria bassiana*	**YFCC 3369**	**Coleoptera: Scarabaeidae**	MN576994	MN576884	MN576938	MW168176	Wang Y. B. et al., 2020; **This study**
*Beauveria blattidicola*	MCA 1727*^T^*	Blattodea: Blattidae	MF416483	MF416640			Kepler et al., 2017
*Beauveria blattidicola*	MCA 1814	Blattodea: Blattidae	MF416484	MF416641			Kepler et al., 2017
*Beauveria brongniartii*	ARSEF 617*^T^*	Coleoptera: Scarabaeidae	HQ880991	HQ880854	HQ880926	HQ880713	Rehner et al., 2011
*Beauveria brongniartii*	**YFCC 3240**	**Coleoptera: Scarabaeidae**	MN576995	MN576885	MN576939	MW168175	Wang Y. B. et al., 2020; **This study**
*Beauveria caledonica*	ARSEF 2567*^T^*	Soil	EF469057	HQ880889	HQ880961	HQ880748	Rehner et al., 2011
*Beauveria caledonica*	**YFCC 7025**	**Coleoptera: Cerambycidae**	MN576997	MN576887	MN576941	MW168178	Wang Y. B. et al., 2020; **This study**
*Beauveria diapheromeriphila*	QCNE 186272*^T^*	Phasmatodea: Diapheromeridae	JQ958610	JX003848			Sanjuan et al., 2014
*Beauveria diapheromeriphila*	QCNE 186714	Phasmatodea: Diapheromeridae	MF416491	MF416648			Kepler et al., 2017
*Beauveria hoplocheli*	Bt116	Coleoptera: Melolonthidae	KC339703	KM453957	KM453966	KM453967	Robène-Soustrade et al., 2015
*Beauveria hoplocheli*	MNHN-RF-06107*^T^*	Coleoptera: Melolonthidae	KC339702	KM453954	KM453963	KM453971	Robène-Soustrade et al., 2015
*Beauveria kipukae*	ARSEF 7032*^T^*	Homoptera: Delphacidae	HQ881005	HQ880875	HQ880947	HQ880734	Rehner et al., 2011
*Beauveria lii*	ARSEF 11741*^T^*	Coleoptera: Coccinellidae	JN689371	JN689374	JN689370	JN689373	Zhang et al., 2012
*Beauveria locustiphila*	TS881	Orthoptera: Romaleidae	JQ958619	JX003847	JX003845		Sanjuan et al., 2014
*Beauveria majiangensis*	GZAC GZU12141*^T^*	Coleoptera: Scarabaeoidea	MG052640	MG052644		MG052639	Chen et al., 2018
*Beauveria majiangensis*	**YFCC 852**	**Coleoptera: Scarabaeidae**	MW168229	MW168195	MW168212	MW168179	**This study**
*Beauveria malawiensis*	ARSEF 7760*^T^*	Coleoptera: Cerambycidae	DQ376246	HQ880897	HQ880969	HQ880756	Rehner et al., 2011
*Beauveria malawiensis*	**YFCC 853**	**Coleoptera: Scarabaeidae**	MW168230	MW168196	MW168213	MW168180	**This study**
*Beauveria medogensis*	2898	Soil	KU994833	KU994835	KU994834	KU994836	Imoulan et al., 2016
*Beauveria medogensis*	**YFCC 854**	**Coleopteran adult**	MW168231	MW168197	MW168214	MW168181	**This study**
*Beauveria peruviensis*	UTRP19 = ARSEF 14196*^T^*	Coleoptera: Curculionidae	MN094781	MN100118		MN094757	Bustamante et al., 2019
*Beauveria peruviensis*	UTRF35	Coleoptera: Curculionidae	MN094771	MN100115		MN094755	Bustamante et al., 2019
*Beauveria polyrhachicola*	**YFCC 859*^T^***	**Hymenoptera: Formicidae**	MW168236	MW168202	MW168219	MW168184	**This study**
*Beauveria polyrhachicola*	**YFCC 867**	**Hymenoptera: Formicidae**	OM373098	OM373099	OM304364	OM373100	**This study**
*Beauveria pseudobassiana*	ARSEF 3405*^T^*	Lepidoptera: Tortricidae	AY531931	HQ880864	HQ880936	HQ880723	Rehner et al., 2011
*Beauveria pseudobassiana*	YFCC 1806007	Coleoptera: Scarabaeidae	MN523553	MN523582	MN523611	MN868289	Wang Y. et al., 2020
*Beauveria rudraprayagi*	MTCC 8017*^T^*	Lepidoptera: Bombycidae	JQ990914	JQ990892	JQ990870	JQ990848	Agrawal et al., 2014
*Beauveria rudraprayagi*	**YFCC 858**	**Lepidopteran larva**	MW168235	MW168201	MW168218	MW168183	**This study**
*Beauveria scarabaeidicola*	ARSEF 1685	Coleoptera: Scarabaeidae	AY531899	HQ880881	HQ880953	HQ880740	Rehner et al., 2011
*Beauveria scarabaeidicola*	ARSEF 5689	Coleoptera: Scarabaeidae	DQ522335	DQ522380	DQ522431	HQ880741	Rehner et al., 2011
*Beauveria scarabaeidicola*	ARSEF 7043	Coleoptera: Scarabaeidae	AY531948	HQ880883	HQ880955	HQ880742	Rehner et al., 2011
*Beauveria scarabaeidicola*	ARSEF 7279	Coleoptera: Scarabaeidae	HQ881009	HQ880885	HQ880957	HQ880744	Rehner et al., 2011
*Beauveria scarabaeidicola*	ARSEF 7281	Coleoptera: Scarabaeidae	HQ881011	HQ880887	HQ880959	HQ880746	Rehner et al., 2011
*Beauveria scarabaeidicola*	**YFCC 865**	**Coleoptera: Scarabaeidae**	MW168243	MW168209	MW168226	MW168191	**This study**
*Beauveria sinensis*	BUB 504	Orthoptera: Grylloidea	MG642895	MG642852	MG642865		Chen et al., 2019
*Beauveria sinensis*	RCEF 3903*^T^*	Lepidoptera: Geometridae	HQ270151	JX524283	JX524284		Chen et al., 2013
*Beauveria songmingensis*	**YFCC 860** *^T^*	**Coleoptera: Scarabaeidae**	MW168238	MW168204	MW168221	MW168186	**This study**
*Beauveria songmingensis*	**YFCC 861**	**Coleoptera: Scarabaeidae**	MW168239	MW168205	MW168222	MW168187	**This study**
*Beauveria staphylinidicola*	ARSEF 5718	Coleoptera: Staphylinidae	EF468776	EF468881		AY883807	Sung et al., 2007
*Beauveria staphylinidicola*	**YFCC 855**	**Coleoptera: Cerambycidae**	MW168232	MW168198	MW168215	MW168182	**This study**
*Beauveria subscarabaeidicola*	**YFCC 863*^T^***	**Coleoptera: Scarabaeidae**	MW168241	MW168207	MW168224	MW168189	**This study**
*Beauveria subscarabaeidicola*	**YFCC 864**	**Coleoptera: Scarabaeidae**	MW168242	MW168208	MW168225	MW168190	**This study**
*Beauveria varroae*	ARSEF 8257*^T^*	Coleoptera: Curculionidae	HQ881002	HQ880872	HQ880944	HQ880731	Rehner et al., 2011
*Beauveria vermiconia*	ARSEF 2922*^T^*	Soil	AY531920	HQ880894	HQ880966	HQ880753	Rehner et al., 2011
*Beauveria yunnanensis*	CCTCC AF 2018010*^T^*	Lepidopteran pupa	MG642900	MG642857	MG642870		Chen et al., 2019
*Beauveria yunnanensis*	YFCC 3105	Coleoptera: Scarabaeidae	MN576999	MN576889	MN576943		Wang Y. B. et al., 2020
*Beauveria yunnanensis*	**YFCC 862**	**Coleoptera: Scarabaeidae**	MW168240	MW168206	MW168223	MW168188	**This study**

*Boldface: data generated in this study. ^T^ex-type material.*

## Materials and Methods

### Soil and Specimen Collection

All the soil samples and the majority of *Beauveria* specimens were collected from Yunnan Province in China. Some specimens were collected from Chiang Rai Province in Thailand. Soil samples and specimens were noted and photographed in the fields, and then carefully placed in plastic containers at low temperature. Afterward, they were carried to the laboratory and stored at 4°C before examination and isolation.

### Fungal Isolation and Culture

*Beauveria* strains were isolated from the soil samples using the *Tenebrio molitor* baiting method (Keyser et al., 2015). Conidia developing on insect cadavers were transplanted onto plates of potato dextrose agar (PDA; potato 200 g/L, dextrose 20 g/L, agar 20 g/L) and cultured at 25°C. Teleomorph specimens were rinsed with tap water, washed with sterile distilled water, and then dried on sterile filter paper. To obtain axenic cultures, white tissue inside the sclerotia of the teleomorph specimens was removed and inoculated onto PDA plates using a sterilized dissecting knife. Colonies of the isolated filamentous fungi appearing in the culture were transferred onto fresh PDA media. The purified fungal strains were maintained in a culture room at 25°C or transferred to PDA slants and stored at 4°C. Specimens were deposited in the Yunnan Herbal Herbarium (YHH) at the Institute of Herb Biotic Resources of Yunnan University. Cultures were stored in the Yunnan Fungal Culture Collection (YFCC) at the Institute of Herb Biotic Resources of Yunnan University.

### Morphological Observations

Specimens were examined using an Olympus SZ61 stereomicroscope (Olympus Corporation, Tokyo, Japan). Cultures on PDA slants were transferred to PDA plates and then incubated at 25°C for 14 days. For morphological evaluation, microscope slides were prepared by placing mycelia from the cultures on PDA medium blocks (5 mm diameter) and then overlaid with a coverslip. Medan dye solution was used to observe asci and ascospores. Other structures were mounted in water. Micro-morphological observations and measurements were performed using a light microscope (CX40, Olympus Corporation, Tokyo, Japan) and a scanning electron microscope (Quanta 200 FEG, FEI Company, Hillsboro, United States). Length to width ratios are given as Q. Mean values for length, width, and Q are indicated by L*^m^*, W*^m^*, and Q*^m^*, respectively.

### DNA Extraction, PCR and Sequencing

Specimens and axenic living cultures were prepared for DNA extraction. Genomic DNA was extracted using the Genomic DNA Purification Kit (Qiagen GmbH, Hilden, Germany) according to the manufacturer’s protocol. The primer pair nrSSU-CoF and nrSSU-CoR was used to amplify a fraction of the nuclear ribosomal small subunit (nr*SSU*) (Wang et al., 2015). Primer pair LR5 and LR0R (Vilgalys and Hester, 1990; Rehner and Samuels, 1994) was used to amplify a fraction of the nuclear ribosomal large subunit (nr*LSU*) and EF1α-EF and EF1α-ER (Bischoff et al., 2006; Sung et al., 2007) for the translation elongation factor 1α (*TEF*). For amplification of the largest and second largest subunits of the RNA polymerase II (*RPB1* and *RPB2*), PCR primer pairs RPB1-5′F/RPB1-5′R and RPB2-5′F/RPB2-5′R (Bischoff et al., 2006; Sung et al., 2007) were employed. The *Bloc* fragment was amplified using primer pair B5.1F/B3.1R (Rehner et al., 2006). All the PCR reactions were performed in a final volume of 50 μL and contained 25 μL of 2 × Taq PCR Master Mix (Tiangen Biotech Co. Ltd, Beijing, China), 0.5 μL of each primer (10 μM), 1 μL of genomic DNA, and 23 μL of RNase-free water. Target gene amplification and sequencing were performed according to the methods described in our previous study (Wang Y. B. et al., 2020).

### Phylogenetic Analyses

Phylogenetic analyses were based on six gene (nr*SSU*, nr*LSU*, *TEF*, *RPB1*, *RPB2*, and *Bloc*) sequences. The sequences were retrieved from GenBank and combined with those generated in our study. Taxon information and GenBank accession numbers were provided in Supplementary Table 1 and Table 1. Sequences were aligned using MAFFT v.7.^1^ After alignment, the sequences of the genes were concatenated. Conflicts among the six genes were tested using PAUP* 4.0b10 (Swofford, 2002). The results revealed that the phylogenetic signals in the six genes were not in conflict. The data partitions were defined for the combined dataset using PartitionFinder V1.1.1 (Lanfear et al., 2012). Phylogenetic analyses were conducted using BI and ML methods employing MrBayes v3.1.2 and RaxML 7.0.3, respectively (Ronquist and Huelsenbeck, 2003; Stamatakis et al., 2008). The BI analysis was run on MrBayes v3.1.2 for five million generations using a GTR+G+I model determined by jModelTest version 2.1.4 (Darriba et al., 2012). GTR+I was selected as the optimal model for ML analysis, and 1,000 rapid bootstrap replicates were performed on the dataset.

The first analysis based on the combined five-gene (nr*SSU*+nr*LSU*+*TEF*+*RPB1*+*RPB2*) dataset was performed using the following taxa: *Akanthomyces*, *Amphichorda*, *Ascopolyporus*, *Beauveria*, *Blackwellomyces*, *Cordyceps*, *Gibellula*, *Hevansia*, *Samsoniella*, and *Simplicillium*. Two taxa of *Trichoderma* were designated as outgroups. The second analysis based on the combined four-gene (*TEF*+*RPB1*+*RPB2*+*Bloc*) sequences was performed using *Beauveria* taxa.

We applied a (phylo-) genetic distance matrix calculation for the combined four-gene (*TEF*+*RPB1*+*RPB2*+*Bloc*) sequences to assess species boundaries in the *B. bassiana* complex and *B. scarabaeidicola* complex (Table 2). The pairwise genetic distances of most *Beauveria* lineages (Supplementary Table 2) were measured based on the Kimura 2-parameter model using MEGA6 software (Tamura et al., 2013).

**TABLE 2:** Genetic distance (p-distances) of species in the *B. bassiana* complex and *B. scarabaeidicola* complex.

Group	Taxa	Marker
		
		*TEF*+*RPB1*+ *RPB2*+*Bloc*
The *B. bassiana* complex	*B. bassiana*—*B. peruviensis*	0.015
	*B. bassiana*—*B. polyrhachicola*	0.019
	*B. bassiana*—*B. rudraprayagi*	0.042
	*B. bassiana*—*B. staphylinidicola*	0.010
	*B. peruviensis*—*B. polyrhachicola*	0.011
	*B. peruviensis*—*B. rudraprayagi*	0.044
	*B. peruviensis*—*B. staphylinidicola*	0.015
	*B. polyrhachicola*—*B. rudraprayagi*	0.045
	*B. polyrhachicola*—*B. staphylinidicola*	0.019
	*B. rudraprayagi*—*B. staphylinidicola*	0.045

The *B. scarabaeidicola* complex	*B. scarabaeidicola*—*B. songmingensis*	0.013
	*B. scarabaeidicola*—*B. subscarabaeidicola*	0.017
	*B. scarabaeidicola*—*B. yunnanensis*	0.014
	*B. songmingensis*—*B. subscarabaeidicola*	0.012
	*B. songmingensis*—*B. yunnanensis*	0.013
	*B. subscarabaeidicola*—*B. yunnanensis*	0.013

### Conidial Viability of *Beauveria* spp. Isolates

A total of 19 *Beauveria* spp. isolates (Table 3) were analyzed for their conidial viability using the method described by Imoulan et al. (2011). The conidial viability of each isolate was confirmed by inoculating three tubes of 3 ml PDB media (potato 200 g/L, dextrose 20 g/L) with 0.1 ml of conidia suspension (3 × 10^6^ conidia/ml). Only isolates with conidial viability greater than 65% were tested for pathogenicity toward *B. mori*, *T. molitor*, and *P. brevitarsis*.

**TABLE 3:** Conidial viability of *Beauveria* spp. isolates used in this study.

Group	Species and isolate	Host/substrate	Location	Conidial viability ± *SE* (%)^#^
The *B. bassiana* complex	*B. bassiana*			
	YFCC 841	Lepidopteran larva	Yunnan province, China	86.33 ± 3.48*^bc^*
	YFCC 842	Hymenoptera: Vespidae	Yunnan province, China	50.86 ± 0.41
	YFCC 843	Lepidoptera: Geometridae	Yunnan province, China	36.80 ± 4.72
	YFCC 844	Soil	Yunnan Province, China	95.00 ± 1.53*^a^*
	YFCC 3369	Coleoptera: Scarabaeidae	Yunnan province, China	84.67 ± 3.76*^bc^*
	*B. polyrhachicola*			
	YFCC 859	Hymenoptera: Formicidae	Chiang Rai Province, Thailand	89.33 ± 0.67*^ab^*
	*B. rudraprayagi*			
	YFCC 858	Lepidopteran larva	Chiang Rai province, Thailand	91.67 ± 1.20*^ab^*
	*B. staphylinidicola*			
	YFCC 845	Coleoptera: Staphylinidae	Yunnan province, China	25.31 ± 0.69
	YFCC 855	Coleoptera: Cerambycidae	Yunnan province, China	81.33 ± 2.33*^c^*

The *B. scarabaeidicola* complex	*B. scarabaeidicola*			
	YFCC 846	Coleoptera: Scarabaeidae	Yunnan province, China	0
	YFCC 847	Coleoptera: Scarabaeidae	Chiang Rai Province, Thailand	65.67 ± 1.20*^d^*
	YFCC 865	Coleoptera: Scarabaeidae	Yunnan province, China	69.00 ± 2.65*^d^*
	*B. songmingensis*			
	YFCC 848	Coleoptera: Scarabaeidae	Yunnan Province, China	42.33 ± 0.67
	YFCC 860	Coleoptera: Scarabaeidae	Yunnan province, China	68.00 ± 3.79*^d^*
	YFCC 861	Coleoptera: Scarabaeidae	Yunnan province, China	0
	*B. subscarabaeidicola*			
	YFCC 863	Coleoptera: Scarabaeidae	Yunnan province, China	18.69 ± 3.25
	YFCC 864	Coleoptera: Scarabaeidae	Yunnan province, China	14.33 ± 2.33
	*B. yunnanensis*			
	YFCC 862	Coleoptera: Scarabaeidae	Yunnan province, China	69.00 ± 3.46*^d^*
	YFCC 3105	Coleoptera: Scarabaeidae	Yunnan province, China	46.67 ± 1.20

*^#^Only isolates with % of conidial viability ≥ 65% were significance tested. Different lowercase letters in the same column indicate significant differences at 5% level.*

### Virulence Assay of *Beauveria* spp. Isolates

A total of 10 *Beauveria* spp. isolates from the *B. bassiana* complex and *B. scarabaeidicola* complex were tested for their pathogenicity to *B. mori* and *T. molitor* larvae in addition to *P. brevitarsis* adults. Conidia for each isolate were obtained from 4-week-old cultures grown on malt extract agar plates, suspended in a sterile aqueous solution of 0.01% Tween 80, and mixed vigorously until homogeneous conidial suspensions were produced. Quantification of the conidia was performed using a hemocytometer under a light microscope at 400 × magnification. All of the suspensions were adjusted to 1 × 10^8^ conidia/ml. The tested insects were individually placed in sterilized rearing boxes and 10 μl of conidial suspension was applied to the surface of each insect. A diet was provided for each insect and renewed as needed. Control groups were treated with the same volume of a sterile aqueous solution of 0.01% Tween 80. The test was replicated three times with 50 insects per replicate. All of the test groups were kept at 25°C under a 12:12 h photoperiod cycle. The numbers of dead insects were recorded every 12 h for a 30 day period, which was used to determine the percentage of mortality. Cadavers were removed, immediately surface-disinfected, and individually placed and maintained in rearing box chambers. Mycelium samples from cadavers were aseptically removed and cultured on PDA for microscope examination, DNA extraction, and *TEF* sequencing to confirm that mortality was caused by the inoculated fungal strain.

## Results

### Sequencing and Phylogenetic Analyses

**FIGURE 1:**
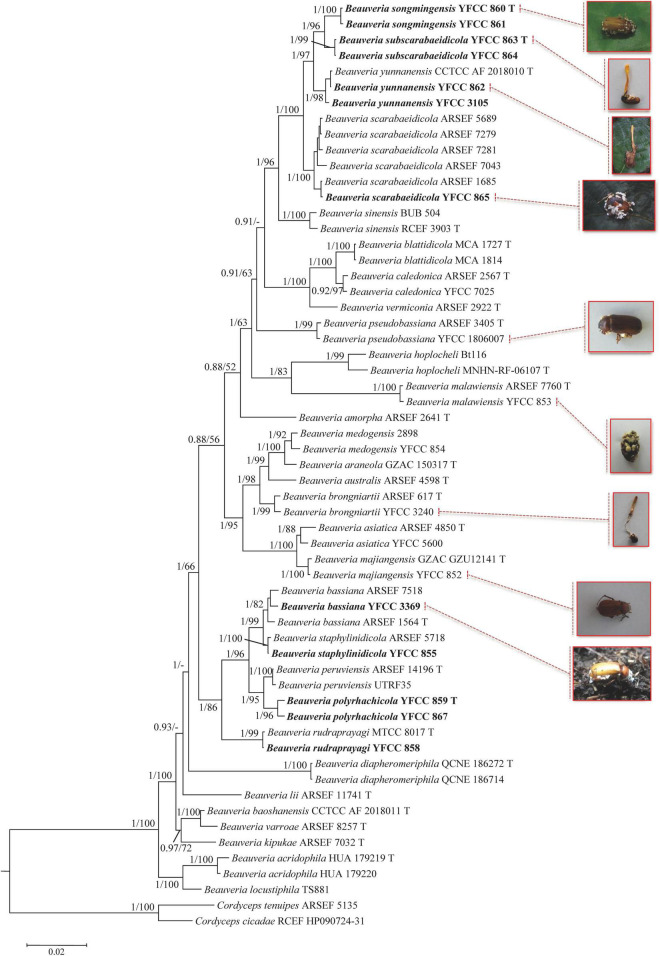
Phylogenetic analysis of *Beauveria* species based on combined partial *TEF*+*RPB1*+*RPB2*+*Bloc* sequences. Statistical support values (≥ 0.5/50%) are shown at the nodes for Bayesian inference (BI) posterior probabilities/maximum likelihood (ML) bootstrap support. Isolates representing ex-type material are marked with “T”. Isolates in bold type are those analyzed in this study.

The combined five-gene dataset included sequences from 123 fungal taxa. The final dataset consisted of 5,001 bp of sequence data (nr*SSU* 1,138 bp, nr*LSU* 910 bp, *TEF* 1,047 bp, *RPB1* 781 bp, and *RPB2* 1,125 bp). Eleven well-supported clades were recognized based on both Bayesian inference (BI) and maximum likelihood (ML) analyses of the combined five-gene dataset of 123 taxa from Cordycipitaceae and *Trichoderma*, which accommodate species of the genera *Akanthomyces*, *Amphichorda*, *Ascopolyporus*, *Beauveria*, *Blackwellomyces*, *Cordyceps*, *Gibellula*, *Hevansia*, *Samsoniella*, *Simplicillium*, and *Trichoderma* (Supplementary Figure 1). The phylogenetic analyses also revealed the species diversity of the *B. bassiana* complex and *B. scarabaeidicola* complex in *Beauveria* clades. This suggested that the groups composed of the *B. bassiana* complex and *B. scarabaeidicola* complex should be genetically composed of at least four species (Supplementary Figure 1). Phylogenetic analyses based on combined partial *TEF*+*RPB1*+*RPB2*+*Bloc* sequences consisting of 59 fungal taxa resolved most *Beauveria* lineages in separate terminal branches (Figure 1). This revealed a similar tree and clustering topology, as shown in Supplementary Figure 1. It was proposed that the two strains YFCC 859 and YFCC 867, which formed a strongly supported clade, might be a new species in the *B. bassiana* complex, named *B. polyrhachicola* (Figure 1). Our analyses also revealed that two newly discovered species, *B. songmingensis* and *B. subscarabaeidicola*, were phylogenetically clustered with *B. yunnanensis* and *B. scarabaeidicola*, but they were clearly distinguished from the latter two by forming two separate clades in the *B. scarabaeidicola* complex (Figure 1 and Supplementary Figure 1). The genetic divergence comparisons showed that the minimum threshold (p-distance) to distinguish genetic species in *Beauveria* was 0.010 for the combined four-gene (*TEF*+*RPB1*+*RPB2*+*Bloc*) sequences, as occurred between *B. araneola* and *B. medogensis*, between *B. asiatica* and *B. majiangensis*, between *B. australis* and *B. brongniartii*, and between *B. bassiana* and *B. staphylinidicola* (Table 2 and Supplementary Table 2). These results also suggested that: (1) the *B. bassiana* complex, which is made up of five species, should include *B. bassiana*, *B. peruviensis*, *B. polyrhachicola*, *B. rudraprayagi*, and *B. staphylinidicola* and (2) the *B. scarabaeidicola* complex should be composed of four species, including *B. scarabaeidicola*, *B. songmingensis*, *B. subscarabaeidicola*, and *B. yunnanensis.*

### Morphological Features

**FIGURE 2:**
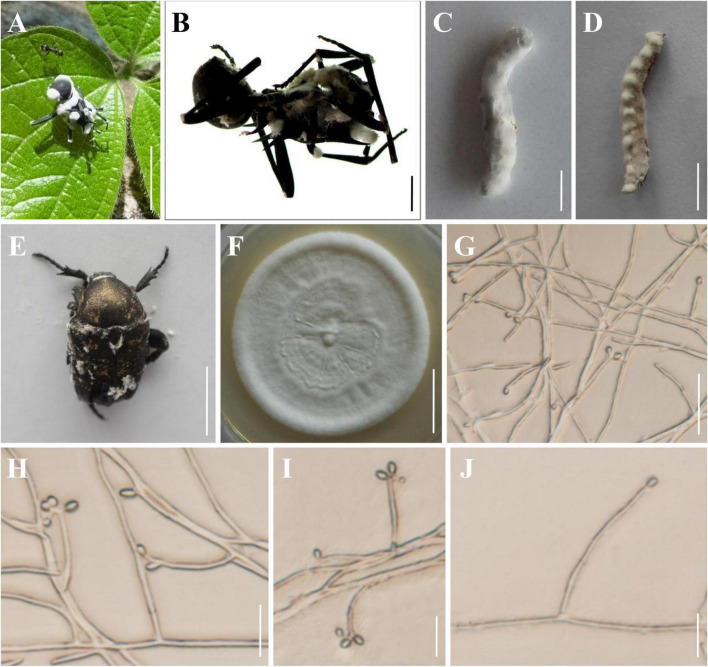
Morphology of *Beauveria polyrhachicola.* **(A,B)** The type specimen (YHH 859). **(C)** *Bombyx mori* larva infected by *B. polyrhachicola*. **(D)** *Tenebrio molitor* larva infected by *B. polyrhachicola*. **(E)** *Protaetia brevitarsis* adult infected by *B. polyrhachicola*. **(F)** Culture character on PDA medium. **(G–J)** Conidiogenous cells and conidia. Scale bars: **(A)** = 10 mm; **(B)** = 2 mm; **(C–E)** = 10 mm; **(F)** = 20 mm; **(G)** = 20 μm; **(H–J)** = 10 μm.

**FIGURE 3:**
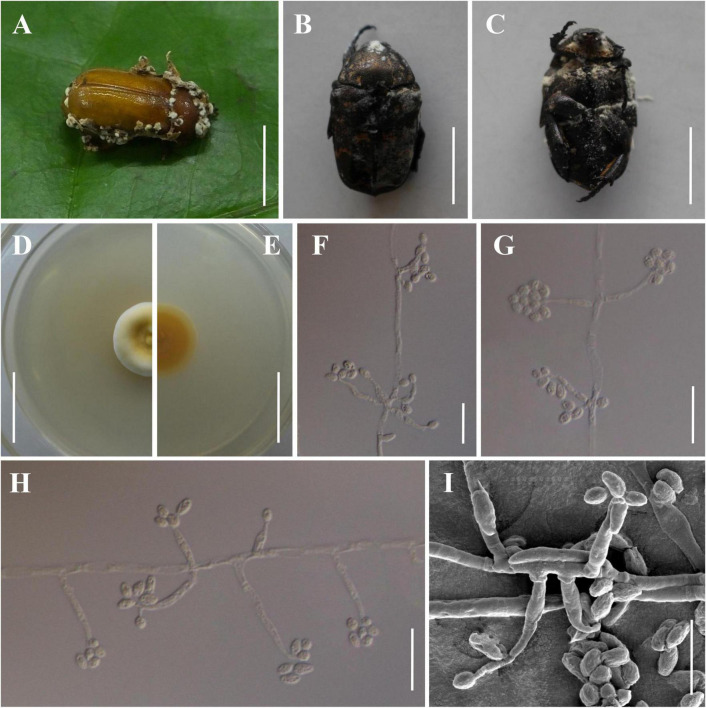
Morphology of *Beauveria songmingensis.* **(A)** The type specimen (YHH 860). **(B,C)** *Protaetia brevitarsis* adults infected by *B. songmingensis*. **(D,E)** Culture character on PDA medium. **(F–I)** Conidiogenous cells and conidia. Scale bars: **(A–C)** = 10 mm; **(D,E)** = 20 mm; **(F–H)** = 20 μm; **(I)** = 10 μm.

**FIGURE 4:**
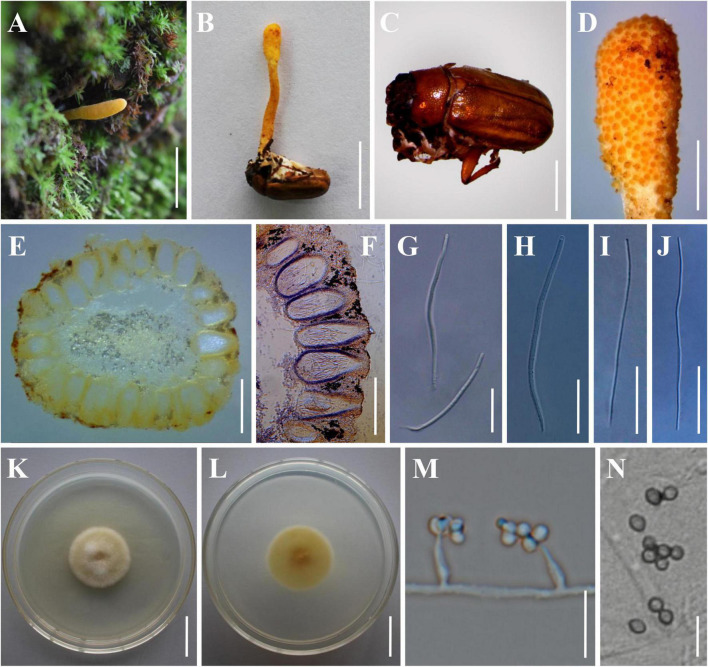
Morphology of *Beauveria subscarabaeidicola.* **(A)** Perithecial stroma as encountered in the field. **(B)** The type specimen (YHH 863). **(C)** The brown host of *B. subscarabaeidicola*. **(D)** Surface of fertile structure of perithecial stroma showing emerging apical parts of immersed perithecia. **(E,F)** Perithecia. **(G,H)** Asci. **(I,J)** Ascospores. **(K,L)** Culture character on PDA medium. **(M,N)** Conidiogenous cells and conidia. Scale bars: **(A,B)** = 10 mm; **(C)** = 5 mm; **(D)** = 2 mm; **(E,F)** = 500 μm; **(G–J)** = 40 μm; **(K,L)** = 20 mm; **(M,N)** = 10 μm.

The morphological characteristics of the three new species as well as photomicrographs of morphological structures are shown in Figures 2–4. The detailed fungal morphological descriptions are provided in the Taxonomy section.

### Conidial Viability of the *Beauveria bassiana* Complex and *Beauveria scarabaeidicola* Complex Isolates

Percentage of conidial germination was used to determine conidial viability. The conidial viability of the *B. bassiana* complex isolates was high, but the highest value ($\bar{x}$ = 95%) was found on isolates of YFCC 844 from soil (see Table 3). The values of the conidial viability of the isolates in the *B. scarabaeidicola* complex were significantly lower than those in the *B. bassiana* complex. Only four *B. scarabaeidicola* complex isolates with conidial viability were greater than 65%, and their conidial viability values were not significantly different (*P* < 0.05).

### Virulence of the *Beauveria bassiana* Complex and *Beauveria scarabaeidicola* Complex Isolates

Ten isolates had conidial viability greater than 65%. These isolates were then selected for pathogenicity tests against *B. mori* and *T. molitor* larvae as well as *P. brevitarsis* adults (Table 4). The *B. bassiana* complex isolates were shown to have great potential for use in the management of various insect pests; by contrast, the *B. scarabaeidicola* complex isolates showed obvious host specificity and low virulence. All tested isolates in the *B. bassiana* complex inflicted mycoses on *B. mori* and *T. molitor* larvae and caused over 80% mortality, whereas those in the *B. scarabaeidicola* complex did not. It was determined that the 10 isolates were pathogenic to *P. brevitarsis* adults but demonstrated different levels of virulence. Like the conidial viability, the mortalities of *P. brevitarsis* adults caused by the *B. scarabaeidicola* complex isolates were significantly lower than those of isolates in the *B. bassiana* complex (*P* < 0.05), strengthening the hypothesis that the virulence of certain entomopathogenic fungi is related to their conidial viability (Butt et al., 1994; Fernandes et al., 2007). Additionally, *B. bassiana* YFCC 844, which was isolated from soil and exhibited the highest conidial viability, showed high virulence against *B. mori* and *T. molitor* larvae, as well as *P. brevitarsis* adults, causing (94.00 ± 1.15)% mortality against *B. mori* larva, (95.33 ± 1.45)% mortality against *T. molitor* larva, and (79.00 ± 1.53)% mortality against *P. brevitarsis* adults (Table 4). Mycelium samples from cadavers were aseptically removed and cultured on PDA. Microscopic examination recovered the same morphological characters of conidiophores and conidia as the inoculated fungal strain. Further, *TEF* sequenced from DNA extracted from recultures of the external mycelium of cadavers were found to match that of inoculated strain perfectly.

**TABLE 4:** The lethal effect of *Beauveria* spp. isolates on *B. mori* and *T. molitor* larvae, and *P. brevitarsis* adult.

Group	Species and isolate	Mortality ± *SE* (%)^#^		
		
		*B. mori* larva	*T. molitor* larva	*P. brevitarsis* adult
The *B. bassiana* complex	*B. bassiana*			
	YFCC 841	89.33 ± 0.88*^a^*	85.00 ± 1.73*^c^*	67.67 ± 1.76*^bc^*
	YFCC 844	94.00 ± 1.15*^a^*	95.33 ± 1.45*^a^*	79.00 ± 1.53*^a^*
	YFCC 3369	81.33 ± 2.03*^b^*	91.33 ± 2.03*^ab^*	74.67 ± 2.60*^ab^*
	*B. polyrhachicola*			
	YFCC 859	82.00 ± 1.53*^b^*	84.00 ± 3.06*^c^*	67.00 ± 3.79*^bc^*
	*B. rudraprayagi*			
	YFCC 858	91.33 ± 2.03*^a^*	87.00 ± 1.15*^bc^*	78.67 ± 2.33*^a^*
	*B. staphylinidicola*			
	YFCC 855	80.67 ± 1.86*^b^*	81.67 ± 1.45*^c^*	62.33 ± 2.03*^c^*

The *B. scarabaeidicola* complex	*B. scarabaeidicola*			
	YFCC 847	0	0	31.33 ± 3.18*^e^*
	YFCC 865	0	0	48.00 ± 5.20*^d^*
	*B. songmingensis*			
	YFCC 860	0	0	49.33 ± 4.70*^d^*
	*B. yunnanensis*			
	YFCC 862	0	0	49.67 ± 5.90*^d^*

*^#^Corrected mortality. Different lowercase letters in the same column indicate significant differences at 5% level.*

## Taxonomy

***Beauveria polyrhachicola* H. Yu & Y. Wang, sp. nov. Figure 2**

MycoBank number 841450.

Etymology: “*polyrhachicola*” refers to the host (*Polyrhachis* sp.).

Sexual morph: Undetermined.

Asexual morph: Colonies on PDA reached 20–38 mm in diameter after 14 days at 25°C, white, circular, velutinous, and closely appressed to the agar surface; reverse yellowish white. Odor indistinct. Vegetative hyphae septate, branched, hyaline, smooth-walled, 1.2–2.3 μm wide. Conidiogenous cells, long cylindrical to long flask shaped, solitary or occurring in dense lateral clusters, base cylindrical to ampulliform and 1.4–3.0 μm wide, sympodially branched neck tapering into a long, slender, denticulate rachis, produced laterally on aerial hyphae or from subtending cells, 11.8–40.9 × 1.4–3.0 μm. Conidia 2.0–3.8 × 1.7–2.6 μm, *Q* = 1.0–1.8 μm (L*^m^* = 2.7 μm, W*^m^* = 2.1 μm, Q*^m^* = 1.3), globose, subglobose, slightly ellipsoid, oblong, or cylindrical, hyaline, aseptate, walls smooth and thin.

Type: Thailand, Chiang Rai Province, Khun Tan District (19.9233°N, 100.3133°E, 396 m above sea level), on an adult worker of *Polyrhachis* sp. emerging from leaf litter on the forest floor, May 2019, collected by Preeyanat Hongthong (holotype: YHH 859; ex-type living culture: YFCC 859).

Distribution: Khun Tan District, Chiang Rai Province, Thailand; Simao District, Yunnan Province, China.

Other material examined: China, Yunnan Province, Puer City, Simao District (22.7113°N, 100.9579°E, 1,360 m above sea level), on an adult worker of *Polyrhachis* sp. emerging from leaf litter on the forest floor, August 26, 2021, Yao Wang (YHH 867, 868; living culture: YFCC 867).

Notes: Regarding phylogenetic relationships, *B. polyrhachicola* forms a distinct lineage in the *B. bassiana* complex, and it is closely related to *B. peruviensis*, *B. staphylinidicola*, *B. bassiana*, and *B. rudraprayagi* (Figure 1). Morphologically, *B. polyrhachicola* is similar to *B. bassiana*, *B. kipukae*, *B. pseudobassiana*, *B. varroae*, and *B. peruviensis* in terms of the shape and size of the conidia (Rehner et al., 2011; Bustamante et al., 2019). However, *B. polyrhachicola* can be distinguished from them by its long conidiogenous cells (11.8–40.9 × 1.4–3.0 μm).

***Beauveria songmingensis* H. Yu & Y. Wang, sp. nov. Figure 3**

MycoBank number 841451.

Etymology: named after the location Songming County where this species was collected.

Sexual morph: Undetermined.

Asexual morph: Colonies on PDA reaching 20–35 mm in diameter after 14 days at 25°C, yellowish white, pale yellow, or light yellow, circular; reverse pale yellow, light yellow, or shades of orange to deep orange. Odor indistinct. Vegetative hyphae septate, branched, hyaline or translucent pale yellow, smooth-walled, 2.2–4.5 μm wide. Conidiogenous cells, cylindrical to long flask shaped, solitary but usually in dense clusters of five or more, base cylindrical to ampulliform and 2.7–5.6 μm wide, apex with an indeterminate 1 μm wide geniculate, denticulate rachis, produced laterally on aerial hyphae or from subtending cells, mostly 9.6–34.1 × 2.7–5.6 μm. Conidia 3.6–6.8 × 2.8–3.9 μm, Q = 1.0–2.0 μm (L*^m^* = 5.6 μm, W*^m^* = 3.4 μm, Q*^m^* = 1.6), subglobose, broadly ellipsoid, ellipsoid, or oblong, hyaline, aseptate, walls smooth and thin.

Type: China, Yunnan Province, Kunming City, Songming County, Dashao Village (25.3924°N, 102.5589°E, 2,700 m above sea level), on an adult of *Pseudosymmachia flavescens* (Coleoptera: Scarabaeidae), August 12, 2018, collected by Yao Wang, (holotype: YHH 860; ex-type living culture: YFCC 860).

Distribution: at present known only in Dashao Village, Songming County, Yunnan Province, China.

Other material examined: China, Yunnan Province, Kunming City, Songming County, Dashao Village (25.3924°N, 102.5589°E, 2,700 m above sea level), on an adult of *Pseudosymmachia* sp. emerging from leaf litter on the forest floor, August 12, 2018, Yao Wang (YHH 848, 861; living culture: YFCC 848, 861).

Notes: Morphologically, *B. songmingensis* resembles the phylogenetically sister species *B. scarabaeidicola* and *B. subscarabaeidicola*. They were found to be parasitic on adult beetles (Coleoptera: Scarabaeidae), and they could be easily recognized by their distinctly yellow colony pigmentation and ellipsoid or oblong conidia. However, our morphological observation revealed a significant difference of conidia sizes between *B. songmingensis* (3.6–6.8 × 2.8–3.9 μm) and *B. scarabaeidicola* (2.5–3.5 × 1.5–2.5 μm). *B. songmingensis* differs from *B. subscarabaeidicola* by its long conidiogenous cells (9.6–34.1 × 2.7–5.6 μm) and large conidia (3.6–6.8 × 2.8–3.9 μm). Both morphological study and phylogenetic analyses of combined *TEF*, *RPB1*, *RPB2*, and *Bloc* sequence data support that this fungus is a distinctive species in the genus *Beauveria*.

***Beauveria subscarabaeidicola* H. Yu, Y. Wang & Q. Fan, sp. nov. Figure 4**

MycoBank number 841452.

Etymology: “*subscarabaeidicola*” refers to morphologically resembling *Beauveria scarabaeidicola* but phylogenetically distinct.

Sexual morph: Stromata solitary, fleshy, pale yellow to orange, arising on adult scarab beetles buried in soil or decayed leaves, 30–45 mm long. Stipes cylindrical to clavate, yellowish white to deep yellow, 1.1–2.0 mm wide. Fertile parts clavate, being slightly wider than and indistinct from the stipes, deep yellow to orange, 5.2–26.0 mm long, 1.4–3.3 mm wide. Perithecia semi-immersed and crowded at the apex of the stromata, ampuliform, pyriform, ovoid to oblong, 265–700 × 180–320 μm (*n* = 50). Asci hyaline, cylindrical, 124.5–257.4 × 3.7–5.2 μm (*n* = 50). Apical caps prominent, hemiglobose, 2.7–3.9 μm wide, 2.4–3.2 μm high (*n* = 50). Ascospores hyaline, filiform, multi-septate, finally breaking into secondary ascospores, 75.6–188.5 × 1.0–1.5 μm (*n* = 30). Secondary ascospores cylindrical, hyaline, 6.9–11.2 × 1.0–1.5 μm (*n* = 50).

Asexual morph: Colonies on PDA reaching 28–42 mm in diameter after 14 days at 25°C, yellowish white, pale yellow, or light yellow, circular; reverse pale yellow, light yellow, or shades of orange to deep orange. Odor indistinct. Vegetative hyphae septate, branched, hyaline or translucent pale yellow, smooth-walled, 1.2–2.5 μm wide. Conidiogenous cells, phialidic, solitary but usually in dense clusters of five or more, base subspherical to ampulliform and 2.8–5.0 μm wide, apex with an indeterminate 1 μm wide geniculate, denticulate rachis, produced laterally on aerial hyphae or from subtending cells mostly 4.8–6.9 × 2.0–4.6 μm. Conidia 2.6–4.2 × 1.9–3.5 μm, Q = 1.0–1.4 μm (L*^m^* = 3.4 μm, W*^m^* = 2.8 μm, Q*^m^* = 1.2), subglobose or broadly ellipsoid, hyaline, aseptate, walls smooth and thin.

Type: China, Yunnan Province, Kunming City, Songming County, Dashao Village (25.2398°N, 102.5617°E, 2,697 m above sea level), on an adult of *Anomala exoleta* (Coleoptera: Scarabaeidae), July 23rd, 2019, collected by Dexiang Tang, (holotype: YHH 863; ex-type living culture: YFCC 863).

Distribution: at present known only from Dashao Village, Songming County, Yunnan Province, China.

Other material examined: China, Yunnan Province, Kunming City, Songming County, Dashao Village (25.2398°N, 102.5617°E, 2,697 m above sea level), on an adult of *Anomala exoleta*, July 23, 2019, Dexiang Tang (YHH 864; living culture: YFCC 864).

Notes: *Beauveria subscarabaeidicola* is practically indistinguishable in morphology to *B. scarabaeidicola*. Our morphological observation revealed no significant differences in the morphological characteristics of teleomorph and anamorph between the two species (Kobayasi and Shimizu, 1976; Rehner et al., 2011; Shrestha et al., 2014). The lack of diagnostic morphological features to distinguish *B. subscarabaeidicola* and *B. scarabaeidicola* was overcome by delimiting the two species using DNA-based methodologies.

## Discussion

It is generally agreed that distinguishing individual *Beauveria* species can be difficult using only morphological characters, as several species in the genus are morphologically cryptic species. In this study, we conducted a comprehensive investigation of the cryptic species diversity of the *B. bassiana* complex and *B. scarabaeidicola* complex. The molecular phylogeny clearly suggested the existence of distinct species in the *B. bassiana* complex and *B. scarabaeidicola* complex that we accordingly propose as new species: *B. polyrhachicola* (Figure 2), *B. songmingensis* (Figure 3), and *B. subscarabaeidicola* (Figure 4). *Beauveria polyrhachicola* is practically indistinguishable in morphology from other members of the *B. bassiana* complex. The shape and size of the conidia and the colony color of *B. polyrhachicola*, among other morphological features, have been observed in *B. bassiana*, *B. rudraprayagi*, *B. staphylinidicola*, and *B. peruviensis* (Rehner et al., 2011; Agrawal et al., 2014; Kepler et al., 2017; Bustamante et al., 2019). In the *B. scarabaeidicola* complex, the macromorphology of *B. scarabaeidicola*, *B. songmingensis*, and *B. subscarabaeidicola* is very similar, and thus species cannot be distinguished visually. The macroscopic and microscopic observations performed during our investigation revealed the extensive overlap in morphological characters and the lack of distinctive phenotypic variation, supporting the notion of cryptic species in a species complex.

At present, multi-locus phylogenetic analyses have gained importance in delimiting the species within the entomopathogenic fungi *Beauveria*. Rehner et al. (2011) divided *B. bassiana s. lat.* and *B. brongniartii s. lat.* into several cryptic species and described six new species based on the Bloc nuclear intergenic region and three nuclear genes encoding elongation factor 1-a (*TEF*), RNA polymerase II largest subunit (*RPB1*), and RNA polymerase II second largest subunit (*RPB2*). Subsequently, more than seven new species and new combinations were confirmed using combined analysis of the four-locus sequence data (Zhang et al., 2012; Chen et al., 2013, 2017, 2018; Agrawal et al., 2014; Robène-Soustrade et al., 2015; Imoulan et al., 2016). In more recent studies, six species were added to the genus based on multilocus (nr*SSU*, nr*LSU*, *TEF*, *RPB1*, and *RPB2*) sequence data: *B. acridophila*, *B. blattidicola*, *B. diapheromeriphila*, *B. locustiphila*, *B. scarabaeidicola*, and *B. staphylinidicola* (Kepler et al., 2017). In this study, we analyzed most species of the newly circumscribed genus *Beauveria* based on phylogenetic inferences of six nuclear molecular markers (nr*SSU*, nr*LSU*, *TEF*, *RPB1*, *RPB2*, and *Bloc*). Phylogenetic analyses based on the five-gene (nr*SSU*, nr*LSU*, *TEF*, *RPB1*, and *RPB2*) dataset and the combined four-gene (*TEF*+*RPB1*+*RPB2*+*Bloc*) sequences produced trees with similar topologies that resolved most *Beauveria* lineages in separate terminal branches (Figure 1 and Supplementary Figure 1). The results of the present work indicate that the first dataset was conducive to determining the phylogenomic relationships between *Beauveria* and its related genera, and the use of the latter was essential to establish robust *Beauveria* species boundaries, particularly the *B. bassiana* complex and *B. scarabaeidicola* complex.

Scarab beetles are leaf and root feeding pests of grasses, grains, sugarcane, strawberry, potato tubers, and young nursery plants (Crocker et al., 1996; Yokoyama et al., 1998). Based on the published literature, there are about six *Beauveria* spp. that parasitize adult scarab beetles: *B. asiatica*, *B. bassiana*, *B. brongniartii*, *B. majiangensis*, *B. pseudobassiana*, and *B. scarabaeidicola* (Rehner et al., 2011; Kepler et al., 2017; Chen et al., 2018; Khonsanit et al., 2020; Wang Y. et al., 2020). Here, we identified an extension of the members to also include *B. malawiensis*, *B. songmingensis*, *B. subscarabaeidicola*, and *B. yunnanensis*, as shown in Figure 1. Chen et al. (2019) emphasized that hosts of *B. yunnanensis* isolates were Lepidoptera pupae. However, our morphological observations of specimens from a type locality of *B. yunnanensis* indicated that their hosts were adult scarab beetles. Moreover, the host of *B. yunnanensis* was not shown in their publication (Chen et al., 2019). It seems that the host of Lepidoptera pupa is doubtful. There is reason to believe that members of the *B. scarabaeidicola* species complex are host-specific.

Not all scarab-killing pathogens are suitable for mycoinsecticide formulations that control scarab beetles. Our data suggested that the *B. scarabaeidicola* complex isolates showed low virulence. In addition, mortalities in *P. brevitarsis* adults caused by the *B. scarabaeidicola* complex isolates were significantly lower than those of isolates in the *B. bassiana* complex. Additional research is needed to determine the effectiveness of other species before future consideration of isolates for biological pest control.

## Conclusion

The *B. bassiana* complex and *B. scarabaeidicola* complex, as special groups in the genus *Beauveria*, are rich in species diversity and have a wide distribution in nature. The *B. bassiana* complex, which is made up of five species, is a cosmopolitan group of soilborne necrotrophic arthropod-pathogenic fungi that have been shown to have great potential for the management of various insect pests. The *B. scarabaeidicola* complex is composed of pathogens specific to scarab beetles, and it is found on leaf litter or buried in soil. Species in this complex are morphologically highly similar and can hardly be distinguished macroscopically. In this study, we reported the discovery and description of three new species: *B. polyrhachicola*, which was found in the *B. bassiana* complex, and *B. songmingensis* and *B. subscarabaeidicola*, which were found in the *B. scarabaeidicola* complex. In addition, 10 species of *Beauveria* were found to be parasitic on scarab beetles. However, not all members are suitable for mycoinsecticide formulations for controlling scarab beetles. Our data suggested that the *B. scarabaeidicola* complex isolates showed obvious low virulence. Additionally, the mortality of *Protaetia brevitarsis* adults caused by the *B. scarabaeidicola* complex isolates was significantly lower than that of isolates in the *B. bassiana* complex.

## Data Availability Statement

The datasets presented in this study can be found in GenBank. The accession numbers can be found in the article/Supplementary Material.

## Author Contributions

YW and HY: conceptualization. YW: methodology, writing—original draft preparation, and formal analysis. YW and QF: software. QF, W-QZ, and DW: validation. YW, DW, D-XT, PH, and HY: investigation. YW, D-XT, and PH: resources. HY: writing—review and editing and funding acquisition. All authors reviewed and approved the final manuscript.

## Conflict of Interest

The authors declare that the research was conducted in the absence of any commercial or financial relationships that could be construed as a potential conflict of interest.

## Publisher’s Note

All claims expressed in this article are solely those of the authors and do not necessarily represent those of their affiliated organizations, or those of the publisher, the editors and the reviewers. Any product that may be evaluated in this article, or claim that may be made by its manufacturer, is not guaranteed or endorsed by the publisher.

## Footnotes

1
http://mafft.cbrc.jp/alignment/server/

## References

[B1] Agrawal Y. Mual P. Shenoy B. D. (2014). Multi-gene genealogies reveal cryptic species *Beauveria rudraprayagi* sp. nov. from India. *Mycosphere* 5 719–736. 10.5943/mycosphere/5/6/3

[B2] Bischoff J. F. Rehner S. A. Humber R. A. (2006). *Metarhizium frigidum* sp. nov.: a cryptic species of *M. anisopliae* and a member of the *M. flavoviride* complex. *Mycologia* 98 737–745. 10.1080/15572536.2006.11832645 17256577

[B3] Bustamante D. E. Oliva M. Leiva S. Mendoza J. E. Bobadilla L. Angulo G. (2019). Phylogeny and species delimitations in the entomopathogenic genus *Beauveria* (Hypocreales, Ascomycota), including the description of *B. peruviensis* sp. nov. *MycoKeys* 58 47–68. 10.3897/mycokeys.58.35764 31565026 PMC6746742

[B4] Butt T. M. Ibrahim L. Ball B. V. Clark S. J. (1994). Pathogenicity of the entomogenous fungi *Metarhizium anisopliae* and *Beauveria bassiana* against crucifer pests and the honey bee. *Biocontrol Sci. Technol.* 4 207–214. 10.1080/09583159409355328

[B5] Chen M. J. Huang B. Li Z. Z. Spatafora J. W. (2013). Morphological and genetic characterisation of *Beauveria sinensis* sp. nov. from China. *Mycotaxon* 124 301–308. 10.5248/124.301 30528588

[B6] Chen W. H. Han Y. F. Liang Z. Q. Jin D. C. (2017). A new araneogenous fungus in the genus *Beauveria* from Guizhou, China. *Phytotaxa* 302 57–64. 10.11646/phytotaxa.302.1.5

[B7] Chen W. H. Liu M. Huang Z. X. Yang G. M. Han Y. F. Liang J. D. (2018). *Beauveria majiangensis*, a new entomopathogenic fungus from Guizhou, China. *Phytotaxa* 333 243–250. 10.11646/phytotaxa.333.2.8

[B8] Chen Z. H. Chen K. Dai Y. D. Zheng Y. Wang Y. B. Yang X. N. (2019). *Beauveria* species diversity in the Gaoligong Mountains of China. *Mycol. Prog.* 18 933–943. 10.1007/s11557-019-01497-z

[B9] Crocker R. L. Rodriguez-del-Bosque L. A. Nailon W. T. Wei X. (1996). Flight periods in Texas of three parasites (Diptera: Pyrgotidae) of adult *Phyllophaga* spp. (Coleoptera: Scarabaeidae) and egg production by *Pyrgota undata*. *Southwest. Entomol.* 21 317–324.

[B10] Darriba D. Taboada G. L. Doallo R. Posada D. (2012). jModelTest 2: more models, new heuristics and parallel computing. *Nat. Methods* 9:772. 10.1038/nmeth.2109 22847109 PMC4594756

[B11] Faria M. R. Wraight S. P. (2007). Mycoinsecticides and mycoacaricides: a comprehensive list with worldwide coverage and international classification of formulation types. *Biol. Control* 43 237–256. 10.1016/j.biocontrol.2007.08.001

[B12] Fernandes ÉK. K. Rangel D. E. N. Moraes ÁM. L. Bittencourt V. R. E. P. Roberts D. W. (2007). Variability in tolerance to UV-B radiation among *Beauveria* spp. isolates. *J. Invertebr. Pathol.* 96 237–243. 10.1016/j.jip.2007.05.007 17610892

[B13] Goettel S. Eilenberg J. Glare T. (2005). “Entomopathogenic fungi and their role in regulation of insect populations,” in *Comprehensive Molecular Insect Science*, eds Gilbert L. B. Latrou K. (Oxford: Elsevier), 361–406.

[B14] Imoulan A. Alaoui A. El Meziane A. (2011). Natural occurrence of soil-borne entomopathogenic fungi in the Moroccan endemic forest of *Argania spinosa* and their pathogenicity to *Ceratitis capitata*. *World J. Microbiol. Biotechnol.* 27 2619–2628. 10.1007/s11274-011-0735-1

[B15] Imoulan A. Wu H. J. Lu W. L. Li Y. Li B. B. Yang R. H. (2016). *Beauveria medogensis* sp. nov., a new fungus of the entomopathogenic genus from China. *J. Invertebr. Pathol.* 139 74–81. 10.1016/j.jip.2016.07.006 27449678

[B16] Kepler R. M. Luangsa-ard J. J. Hywel-Jones N. L. Quandt C. A. Sung G. H. Rehner S. A. (2017). A phylogenetically-based nomenclature for Cordycipitaceae (Hypocreales). *IMA Fungus* 8 335–353. 10.5598/imafungus.2017.08.02.08 29242779 PMC5729716

[B17] Kepler R. M. Sung G. H. Ban S. Nakagiri A. Chen M. J. Huang B. (2012). New teleomorph combinations in the entomopathogenic genus *Metacordyceps*. *Mycologia* 104 182–197. 10.3852/11-070 22067304

[B18] Keyser C. A. De Fine Licht H. H. Steinwender B. M. Meyling N. V. (2015). Diversity within the entomopathogenic fungal species *Metarhizium flavoviride* associated with agricultural crops in Denmark. *BMC Microbiol.* 15:249. 10.1186/s12866-015-0589-z 26519342 PMC4628438

[B19] Khonsanit A. Luangsa-ard J. J. Thanakitpipattana D. Noisripoom W. Chaitika T. Kobmoo N. (2020). Cryptic diversity of the genus *Beauveria* with a new species from Thailand. *Mycol. Prog.* 19 291–315. 10.1007/s11557-020-01557-9

[B20] Kobayasi Y. Shimizu D. (1976). The genus *Cordyceps* and its allies from New Guinea. *Bull. Natl. Sci. Mus. Ser. B* 2 133–151.

[B21] Lanfear R. Calcott B. Ho S. Y. W. Guindon S. (2012). Partitionfinder: combined selection of partitioning schemes and substitution models for phylogenetic analyses. *Mol. Biol. Evol.* 29 1695–1701. 10.1093/molbev/mss020 22319168

[B22] Li Z. Z. Huang B. Chen M. J. Wang B. Fan M. Z. (2011). Studies on the genus *Beauveria* in molecular era. *Mycosystema* 30 823–835.

[B23] Luo Z. Zhang T. Liu P. Bai Y. Chen Q. Zhang Y. (2018). The *Beauveria bassiana* Gas 3 β-glucanosyltransferase contributes to fungal adaptation to extreme alkaline conditions. *Appl. Environ. Microbiol.* 84:e01086-18. 10.1128/AEM.01086-18 29802184 PMC6052264

[B24] McKinnon A. C. Glare T. R. Ridgway H. J. Mendoza-Mendoza A. Holyoake A. Godsoe W. K. (2018). Detection of the entomopathogenic fungus *Beauveria bassiana* in the rhizosphere of wound-stressed *Zea mays* plants. *Front. Microbiol.* 9:1161. 10.3389/fmicb.2018.01161 29942287 PMC6004820

[B25] Ownley B. H. Gwinn K. D. Vega F. E. (2010). Endophytic fungal entomopathogens with activity against plant pathogens: ecology and evolution. *Biocontrol* 55 113–128. 10.1007/s10526-009-9241-x

[B26] Rehner S. A. Minnis A. M. Sung G. H. Luangsa-ard J. J. Devotto L. Humber R. A. (2011). Phylogeny and systematics of the anamorphic, entomopathogenic genus *Beauveria*. *Mycologia* 103 1055–1073. 10.3852/10-302 21482632

[B27] Rehner S. A. Posada F. Buckley E. P. Infante F. Castillo A. Vega F. E. (2006). Phylogenetic origins of African and Neotropical *Beauveria bassiana s.l.* pathogens of the coffee berry borer, *Hypothenemus hampei*. *J. Invertebr. Pathol.* 93 11–21. 10.1016/j.jip.2006.04.005 16806258

[B28] Rehner S. A. Samuels G. J. (1994). Taxonomy and phylogeny of *Gliocladium* analysed from nuclear large subunit ribosomal DNA sequences. *Mycol. Res.* 98 625–634. 10.1016/S0953-7562(09)80409-7

[B29] Robène-Soustrade I. Jouen E. Pastou D. Payet-Hoarau M. Goble T. Linderme D. (2015). Description and phylogenetic placement of *Beauveria hoplocheli* sp. nov. used in the biological control of the sugarcane white grub, *Hoplochelus marginalis*, on Reunion Island. *Mycologia* 107 1221–1232. 10.3852/14-344 26297783

[B30] Ronquist F. Huelsenbeck J. P. (2003). MrBayes 3: Bayesian phylogenetic inference under mixed models. *Bioinformatics* 19 1572–1574. 10.1093/bioinformatics/btg180 12912839

[B31] Sanjuan T. Tabima J. Restrepo S. Læssøe T. Spatafora J. W. Franco-Molano A. E. (2014). Entomopathogens of Amazonian stick insects and locusts are members of the *Beauveria* species complex (*Cordyceps* sensu stricto). *Mycologia* 106 260–275. 10.3852/13-020 24782494

[B32] Shrestha B. Hyun M. W. Oh J. Han J. G. Lee T. H. Cho J. Y. (2014). Molecular evidence of a teleomorph-anamorph connection between *Cordyceps scarabaeicola* and *Beauveria sungii* and its implication for the systematics of *Cordyceps* sensu stricto. *Mycoscience* 55 231–239. 10.1016/j.myc.2013.09.004

[B33] Stamatakis A. Hoover P. Rougemont J. (2008). A rapid bootstrap algorithm for the RAxML web servers. *Syst. Biol.* 57 758–771. 10.1080/10635150802429642 18853362

[B34] Sung G. H. Hywel-Jones N. L. Sung J. M. Luangsa-ard J. J. Shrestha B. Spatafora J. W. (2007). Phylogenetic classification of *Cordyceps* and the clavicipitaceous fungi. *Stud. Mycol.* 57 5–59. 10.3114/sim.2007.57.01 18490993 PMC2104736

[B35] Swofford D. L. (2002). *PAUP*. Phylogenetic Analysis Using Parsimony (*and Other Methods), Version 4.0b10.* Sunderland, MA: Sinauer.

[B36] Tamura K. Stecher G. Peterson D. Filipski A. Kumar S. (2013). MEGA6: molecular evolutionary genetics analysis version 6.0. *Mol. Biol. Evol.* 30 2725–2729. 10.1093/molbev/mst197 24132122 PMC3840312

[B37] Vilgalys R. Hester M. (1990). Rapid genetic identification and mapping of enzymatically amplified ribosomal DNA from several *Cyptococcus* species. *J. Bacteriol.* 172 4238–4246. 10.1128/jb.172.8.4238-4246.1990 2376561 PMC213247

[B38] Wang Y. Tang D. X. Duan D. E. Wang Y. B. Yu H. (2020). Morphology, molecular characterization, and virulence of *Beauveria pseudobassiana* isolated from different hosts. *J. Invertebr. Pathol.* 172:107333. 10.1016/j.jip.2020.107333 32001215

[B39] Wang Y. B. Wang Y. Fan Q. Duan D. E. Zhang G. D. Dai R. Q. (2020). Multigene phylogeny of the family Cordycipitaceae (Hypocreales): new taxa and the new systematic position of the Chinese cordycipitoid fungus *Paecilomyces hepiali*. *Fungal Divers.* 103 1–46. 10.1007/s13225-020-00457-3

[B40] Wang Y. B. Yu H. Dai Y. D. Wu C. K. Zeng W. B. Yuan F. (2015). *Polycephalomyces agaricus*, a new hyperparasite of *Ophiocordyceps* sp. infecting melolonthid larvae in southwestern China. *Mycol. Prog.* 14:70. 10.1007/s11557-015-1090-7

[B41] Yokoyama T. Hasegawa M. Fujiie A. Sawada M. Noguchi K. (1998). Microbial control of scarab beetle larvae by a formulation of *Metarhizium anisopliae* (Deuteromycotina: Hyphomycetes) in a sweet potato field. *Appl. Entomol. Zool.* 33 215–218. 10.1303/aez.33.215 33922110

[B42] Zhang S. L. He L. M. Chen X. Huang B. (2012). *Beauveria lii* sp. nov. isolated from *Henosepilachna vigintioctopunctata*. *Mycotaxon* 121 199–206. 10.5248/121.199

[B43] Zimmermann G. (2007). Review on safety of the entomopathogenic fungi *Beauveria bassiana* and *Beauveria brongniartii*. *Biocontrol Sci. Technol.* 17 553–596. 10.1080/09583150701309006

