# Supplementary material for: Species Diversity and Virulence Potential of the Beauveria bassiana Complex and Beauveria scarabaeidicola Complex
Source: Front Microbiol. 2022 Mar 4;13:841604. doi: 10.3389/fmicb.2022.841604 (PMC8934399; doi:10.3389/fmicb.2022.841604)
Supplement: Supplementary file 1 [file Data_Sheet_1.zip › Table S1.docx]

**TABLE S1** Specimen information and GenBank accession numbers for sequences used in this study

| **Taxon** | **Voucher information** | **Host/Substrate** | **GenBank accession number** | | | | |
| --- | --- | --- | --- | --- | --- | --- | --- |
|  |  |  | **nr*SSU*** | **nr*LSU*** | ***TEF*** | ***RPB1*** | ***RPB2*** |
| *Trichoderma deliquescens* | ATCC 208838 | On decorticated conifer wood | AF543768 | AF543791 | AF543781 | AY489662 | DQ522446 |
| *Trichoderma stercorarium* | ATCC 62321 | Cow dung | AF543769 | AF543792 | AF543782 | AY489633 | EF469103 |
| *Akanthomyces attenuatus* | CBS 402.78 | Leaf litter | AF339614 | AF339565 | EF468782 | EF468888 | EF468935 |
| *Akanthomyces coccidioperitheciatus* | NHJ 6709 | Araneae | EU369110 | EU369042 | EU369025 | EU369067 | EU369086 |
| *Akanthomyces dipterigenus* | CBS 126.27 | Hemiptera: Monophlebidae | AF339605 | AF339556 | KM283820 | KR064300 | KR064303 |
| *Akanthomyces lecanii* | CBS 101247 | Hemiptera: Coccidae | AF339604 | AF339555 | DQ522359 | DQ522407 | DQ522466 |
| *Akanthomyces muscarius* | CBS 143.62 | Hemiptera: Aleyrodidae | KM283774 | KM283798 | KM283821 | KM283841 | KM283863 |
| *Akanthomyces sabanensis* | ANDES-F 1024 | Hemiptera: Coccidae | KC633251 | KC875225 | KC633266 |  | KC633249 |
| *Akanthomyces sulphureus* | TBRC 7248 | Araneae |  | MF140722 | MF140843 | MF140787 | MF140812 |
| *Akanthomyces tuberculatus* | BCC 16819 | Lepidopteran adult | MF416600 | MF416546 | MF416490 | MF416647 | MF416444 |
| *Akanthomyces waltergamsii* | TBRC 7251 | Araneae |  | MF140713 | MF140833 | MF140781 | MF140805 |
| *Akanthomyces waltergamsii* | TBRC 7252 | Araneae |  | MF140714 | MF140834 | MF140782 | MF140806 |
| ***Amphichorda felina*** | **YFCC 850** | **Bird droppings** | **MW181774** | **MW173986** | **MW168227** | **MW168193** | **MW168210** |
| ***Amphichorda felina*** | **YFCC 851** | **Bird droppings** | **MW181775** | **MW173987** | **MW168228** | **MW168194** | **MW168211** |
| *Amphichorda guana* | CGMCC 3.17908 | Bat guano | KY883262 | KU746711 | KX855211 | KY883202 | KY883228 |
| *Amphichorda guana* | CGMCC 3.17909 | Bat guano | KY883263 | KU746712 | KX855212 | KY883203 |  |
| *Ascopolyporus polychrous* | P.C. 546 | Plant |  | DQ118737 | DQ118745 | DQ127236 |  |
| *Ascopolyporus villosus* | ARSEF 6355 | Plant |  | AY886544 | DQ118750 | DQ127241 |  |
| *Beauveria acridophila* | HUA 179219 | Orthoptera: Acrididae |  | JQ895541 | JQ958613 | JX003857 | JX003841 |
| *Beauveria acridophila* | HUA 179220 | Orthoptera: Acrididae | JQ895527 | JQ895536 | JQ958614 | JX003852 | JX003842 |
| *Beauveria amorpha* | ARSEF 2641 | Hymenoptera: Formicidae |  |  | AY531917 | HQ880880 | HQ880952 |
| *Beauveria araneola* | GZAC 150317 | Araneae |  |  | KT961699 | KT961701 |  |
| *Beauveria asiatica* | ARSEF 4850 | Coleoptera: Cerambycidae |  |  | AY531937 | HQ880859 | HQ880931 |
| ***Beauveria asiatica*** | **YFCC 5600** | **Coleoptera: Cerambycidae** | **MN576770** | **MN576826** | **MN576996** | **MN576886** | **MN576940** |
| *Beauveria australis* | ARSEF 4598 | Soil |  |  | HQ880995 | HQ880861 | HQ880933 |
| *Beauveria baoshanensis* | CCTCC AF 2018011 | Coleoptera: Chrysomelidae | MG642882 | MG642840 | MG642897 | MG642854 | MG642867 |
| *Beauveria bassiana* | ARSEF 300 | Hemiptera: Lygaeidae |  |  | AY531924 | HQ880831 | HQ880903 |
| *Beauveria bassiana* | ARSEF 1040 | Lepidoptera: Bombycidae |  |  | AY531881 | HQ880830 | HQ880902 |
| *Beauveria bassiana* | ARSEF 1478 | Hemiptera: Pentatomidae |  |  | AY531890 | HQ880836 | HQ880908 |
| *Beauveria bassiana* | ARSEF 1564 | Lepidoptera: Arctiidae |  |  | HQ880974 | HQ880833 | HQ880905 |
| *Beauveria bassiana* | ARSEF 1811 | Coleoptera: Curculionidae |  |  | AY531901 | HQ880837 | HQ880909 |
| *Beauveria bassiana* | ARSEF 7518 | Hymenoptera: Pamphiliidae |  |  | HQ880975 | HQ880834 | HQ880906 |
| ***Beauveria bassiana*** | **YFCC 3369** | **Coleoptera: Scarabaeidae** | **MN576768** | **MN576824** | **MN576994** | **MN576884** | **MN576938** |
| *Beauveria blattidicola* | MCA 1727 | Blattodea: Blattidae | MF416593 | MF416539 | MF416483 | MF416640 |  |
| *Beauveria blattidicola* | MCA 1814 | Blattodea: Blattidae | MF416594 | MF416540 | MF416484 | MF416641 |  |
| *Beauveria brongniartii* | ARSEF 617 | Coleoptera: Scarabaeidae | AB027335 | AB027381 | HQ880991 | HQ880854 | HQ880926 |
| ***Beauveria brongniartii*** | **YFCC 3240** | **Coleoptera: Scarabaeidae** | **MN576769** | **MN576825** | **MN576995** | **MN576885** | **MN576939** |
| *Beauveria caledonica* | ARSEF 2567 | Soil | AF339570 | AF339520 | EF469057 | HQ880889 | HQ880961 |
| ***Beauveria caledonica*** | **YFCC 7025** | **Coleoptera: Cerambycidae** | **MN576771** | **MN576827** | **MN576997** | **MN576887** | **MN576941** |
| *Beauveria diapheromeriphila* | QCNE 186272 | Phasmatodea: Diapheromeridae | JQ895530 | JQ895534 | JQ958610 | JX003848 |  |
| *Beauveria diapheromeriphila* | QCNE 186714 | Phasmatodea: Diapheromeridae | MF416601 | MF416547 | MF416491 | MF416648 |  |
| *Beauveria hoplocheli* | Bt116 | Coleoptera: Melolonthidae |  |  | KC339703 | KM453957 | KM453966 |
| *Beauveria hoplocheli* | MNHN-RF-06107 | Coleoptera: Melolonthidae |  |  | KC339702 | KM453954 | KM453963 |
| *Beauveria kipukae* | ARSEF 7032 | Homoptera: Delphacidae |  |  | HQ881005 | HQ880875 | HQ880947 |
| *Beauveria lii* | ARSEF 11741 | Coleoptera: Coccinellidae |  |  | JN689371 | JN689374 | JN689370 |
| *Beauveria locustiphila* | TS881 | Orthoptera: Romaleidae | JQ895525 | JQ895535 | JQ958619 | JX003847 | JX003845 |
| *Beauveria majiangensis* | GZAC GZU12141 | Coleoptera: Scarabaeoidea |  |  | MG052640 | MG052644 |  |
| ***Beauveria majiangensis*** | **YFCC 852** | **Hemiptera: Pentatomidae** | **MW181776** | **MW173988** | **MW168229** | **MW168195** | **MW168212** |
| *Beauveria malawiensis* | ARSEF 7760 | Coleoptera: Cerambycidae |  |  | DQ376246 | HQ880897 | HQ880969 |
| ***Beauveria malawiensis*** | **YFCC 853** | **Coleoptera: Scarabaeoidea** | **MW181777** | **MW173989** | **MW168230** | **MW168196** | **MW168213** |
| *Beauveria medogensis* | 2898 | Soil |  |  | KU994833 | KU994835 | KU994834 |
| ***Beauveria medogensis*** | **YFCC 854** | **Coleopteran adult** | **MW181778** | **MW173990** | **MW168231** | **MW168197** | **MW168214** |
| *Beauveria peruviensis* | ARSEF 14196 | Coleoptera: Curculionidae |  |  | MN094781 | MN100118 |  |
| *Beauveria peruviensis* | UTRF35 | Coleoptera: Curculionidae |  |  | MN094771 | MN100115 |  |
| ***Beauveria polyrhachicola*** | **YFCC 859** | **Hymenoptera: Formicidae** | **MW181783** | **MW173995** | **MW168236** | **MW168202** | **MW168219** |
| ***Beauveria polyrhachicola*** | **YHH 859** | **Hymenoptera: Formicidae** | **MW181784** | **MW173996** | **MW168237** | **MW168203** | **MW168220** |
| *Beauveria pseudobassiana* | ARSEF 3405 | Lepidoptera: Tortricidae |  |  | AY531931 | HQ880864 | HQ880936 |
| ***Beauveria pseudobassiana*** | **YFCC 1806007** | **Coleoptera: Scarabaeidae** | **MN523495** | **MN523524** | **MN523553** | **MN523582** | **MN523611** |
| *Beauveria scarabaeidicola* | ARSEF 1685 | Coleoptera: Scarabaeidae |  |  | AY531899 | HQ880881 | HQ880953 |
| *Beauveria scarabaeidicola* | ARSEF 5689 | Coleoptera: Scarabaeidae | AF339574 | AF339524 | DQ522335 | DQ522380 | DQ522431 |
| *Beauveria scarabaeidicola* | ARSEF 7043 | Coleoptera: Scarabaeidae |  |  | AY531948 | HQ880883 | HQ880955 |
| *Beauveria scarabaeidicola* | ARSEF 7279 | Coleoptera: Scarabaeidae |  |  | HQ881009 | HQ880885 | HQ880957 |
| *Beauveria scarabaeidicola* | ARSEF 7281 | Coleoptera: Scarabaeidae |  |  | HQ881011 | HQ880887 | HQ880959 |
| *Beauveria sinensis* | BUB 504 | Orthoptera: Grylloidea | MG642880 | MG642838 | MG642895 | MG642852 | MG642865 |
| *Beauveria sinensis* | RCEF 3903 | Lepidoptera: Geometridae |  |  | HQ270151 | JX524283 | JX524284 |
| ***Beauveria songmingensis*** | **YFCC 860** | **Coleoptera: Scarabaeidae** | **MW181785** | **MW173997** | **MW168238** | **MW168204** | **MW168221** |
| ***Beauveria songmingensis*** | **YFCC 861** | **Coleoptera: Scarabaeidae** | **MW181786** | **MW173998** | **MW168239** | **MW168205** | **MW168222** |
| *Beauveria staphylinidicola* | ARSEF 5718 | Coleoptera: Staphylinidae | EF468981 | EF468836 | EF468776 | EF468881 |  |
| ***Beauveria staphylinidicola*** | **YFCC 855** | **Coleoptera: Cerambycidae** | **MW181779** | **MW173991** | **MW168232** | **MW168198** | **MW168215** |
| ***Beauveria subscarabaeidicola*** | **YFCC 863** | **Coleoptera: Scarabaeidae** | **MW181788** | **MW174000** | **MW168241** | **MW168207** | **MW168224** |
| ***Beauveria subscarabaeidicola*** | **YFCC 864** | **Coleoptera: Scarabaeidae** | **MW181789** | **MW174001** | **MW168242** | **MW168208** | **MW168225** |
| *Beauveria varroae* | ARSEF 8257 | Coleoptera: Curculionidae |  |  | HQ881002 | HQ880872 | HQ880944 |
| *Beauveria vermiconia* | ARSEF 2922 | Soil |  |  | AY531920 | HQ880894 | HQ880966 |
| *Beauveria yunnanensis* | CCTCC AF 2018010 | Lepidopteran pupa | MG642885 | MG642843 | MG642900 | MG642857 | MG642870 |
| ***Beauveria yunnanensis*** | **YFCC 862** | **Coleoptera: Scarabaeidae** | **MW181787** | **MW173999** | **MW168240** | **MW168206** | **MW168223** |
| ***Beauveria yunnanensis*** | **YFCC 3105** | **Coleoptera: Scarabaeidae** | **MN576773** | **MN576829** | **MN576999** | **MN576889** | **MN576943** |
| *Blackwellomyces cardinalis* | OSC 93609 | Lepidoptera: Tineidae | AY184973 | AY184962 | DQ522325 | DQ522370 | DQ522422 |
| *Blackwellomyces cardinalis* | OSC 93610 | Lepidoptera: Tineidae | AY184974 | AY184963 | EF469059 | EF469088 | EF469106 |
| *Blackwellomyces pseudomilitaris* | BCC 1919 | Lepidopteran larva | MF416588 | MF416534 | MF416478 |  | MF416440 |
| *Blackwellomyces pseudomilitaris* | BCC 2091 | Lepidopteran larva | MF416589 | MF416535 | MF416479 |  | MF416441 |
| *Cordyceps amoene-rosea* | CBS 107.73 | Coleopteran pupa | AY526464 | MF416550 | MF416494 | MF416651 | MF416445 |
| *Cordyceps bifusispora* | EFCC 5690 | Lepidopteran pupa | EF468952 | EF468806 | EF468746 | EF468854 | EF468909 |
| *Cordyceps bifusispora* | EFCC 8260 | Lepidopteran pupa | EF468953 | EF468807 | EF468747 | EF468855 | EF468910 |
| *Cordyceps blackwelliae* | TBRC 7256 | Coleopteran larva |  | MF140702 | MF140822 | MF140771 | MF140795 |
| ***Cordyceps blackwelliae*** | **YFCC 856** | **Lepidopteran larva** | **MW181780** | **MW173992** | **MW168233** | **MW168199** | **MW168216** |
| *Cordyceps caloceroides* | MCA 2249 | Araneae | MF416578 | MF416525 | MF416470 | MF416632 |  |
| *Cordyceps cateniobliqua* | CBS 153.83 | Lepidoptera: Tortricidae | AY526466 |  | JQ425688 |  | MG665236 |
| ***Cordyceps cateniobliqua*** | **YFCC 3367** | **Coleopteran adult** | **MN576765** | **MN576821** | **MN576991** | **MN576881** | **MN576935** |
| *Cordyceps chiangdaoensis* | TBRC 7274 | Coleoptera |  | MF140732 | KT261403 |  |  |
| ***Cordyceps chiangdaoensis*** | **YFCC 857** | **Coleoptera: Elateridae** | **MW181781** | **MW173993** | **MW168234** | **MW168200** | **MW168217** |
| *Cordyceps cicadae* | RCEF HP090724-31 | Hemiptera: Cicadidae | MF416605 | MF416552 | MF416496 | MF416653 | MF416447 |
| *Cordyceps coleopterorum* | CBS 110.73 | Coleopteran larva | JF415965 | JF415988 | JF416028 | JN049903 | JF416006 |
| *Cordyceps exasperata* | MCA 2288 | Lepidopteran larva | MF416592 | MF416538 | MF416482 | MF416639 |  |
| *Cordyceps farinosa* | CBS 111113 | Unknown | AY526474 | MF416554 | MF416499 | MF416656 | MF416450 |
| *Cordyceps fumosorosea* | CBS 244.31 | Butter | MF416609 | MF416557 | MF416503 | MF416660 | MF416454 |
| ***Cordyceps fumosorosea*** | **YFCC 4561** | **Lepidoptera** | **MN576761** | **MN576817** | **MN576987** | **MN576877** | **MN576931** |
| *Cordyceps javanica* | CBS 134.22 | Coleoptera | MF416610 | MF416558 | MF416504 | MF416661 | MF416455 |
| *Cordyceps javanica* | TBRC 7259 | Lepidoptera |  | MF140711 | MF140831 | MF140780 | MF140804 |
| ***Cordyceps javanica*** | **YFCC 3368** | **Lepidoptera** | **MN576767** | **MN576823** | **MN576993** | **MN576883** | **MN576937** |
| *Cordyceps kyusyuensis* | EFCC 5886 | Lepidopteran pupa | EF468960 | EF468813 | EF468754 | EF468863 | EF468917 |
| ***Cordyceps militaris*** | **YFCC 6587** | **Lepidopteran pupa** | **MN576762** | **MN576818** | **MN576988** | **MN576878** | **MN576932** |
| *Cordyceps ninchukispora* | EGS 38.165 | Plant(*Beilschmiedia erythrophloia*) | EF468991 | EF468846 | EF468795 | EF468900 |  |
| *Cordyceps ninchukispora* | EGS 38.166 | Plant(*Beilschmiedia erythrophloia*) | EF468992 | EF468847 | EF468794 | EF468901 |  |
| *Cordyceps oncoperae* | ARSEF 4358 | Lepidoptera: Hepialidae | AF339581 | AF339532 | EF468785 | EF468891 | EF468936 |
| *Cordyceps polyarthra* | MCA 996 | Lepidoptera | MF416597 | MF416543 | MF416487 | MF416644 |  |
| *Cordyceps polyarthra* | MCA 1009 | Lepidoptera | MF416598 | MF416544 | MF416488 | MF416645 |  |
| *Cordyceps pruinosa* | ARSEF 5413 | Lepidoptera: Limacodidae | AY184979 | AY184968 | DQ522351 | DQ522397 | DQ522451 |
| *Cordyceps rosea* | spat 09-053 | Lepidopteran larva | MF416590 | MF416536 | MF416480 | MF416637 | MF416442 |
| *Cordyceps tenuipes* | ARSEF 5135 | Lepidopteran pupa | MF416612 | JF415980 | JF416020 | JN049896 | JF416000 |
| *Gibellula leiopus* | BCC 16025 | Araneae | MF416602 | MF416548 | MF416492 | MF416649 |  |
| *Gibellula longispora* | NHJ 12014 | Araneae | EU369098 |  | EU369017 | EU369055 | EU369075 |
| *Gibellula pulchra* | NHJ 10808 | Araneae | EU369099 | EU369035 | EU369018 | EU369056 | EU369076 |
| *Hevansia arachnophilus* | NHJ 10469 | Araneae | EU369090 | EU369031 | EU369008 | EU369047 |  |
| *Hevansia cinereus* | NHJ 3510 | Araneae | EU369091 |  | EU369009 | EU369048 | EU369070 |
| *Hevansia nelumboides* | BCC 41864 | Araneae | JN201863 | JN201873 | JN201867 |  |  |
| *Hevansia novoguineensis* | NHJ 11923 | Araneae | EU369095 | EU369032 | EU369013 | EU369052 | EU369072 |
| *Samsoniella alboaurantium* | CBS 240.32 | Lepidopteran pupa | JF415958 | JF415979 | JF416019 | JN049895 | JF415999 |
| *Samsoniella alboaurantium* | CBS 262.58 | Soil | AB023943 | AB080087 | MF416497 | MF416654 | MF416448 |
| *Samsoniella aurantia* | TBRC 7271 | Lepidoptera |  | MF140728 | MF140846 | MF140791 | MF140818 |
| *Samsoniella inthanonensis* | TBRC 7915 | Lepidopteran pupa |  | MF140725 | MF140849 | MF140790 | MF140815 |
| *Simplicillium formicae* | MFLUCC 18-1379 | Hymenoptera: Formicidae | MK765046 | MK766512 | MK926451 | MK882623 |  |
| *Simplicillium lamellicola* | CBS 116.25 | Fungi (*Agaricus bisporus*) | AF339601 | AF339552 | DQ522356 | DQ522404 | DQ522462 |
| *Simplicillium lanosoniveum* | CBS 704.86 | Fungi (*Hemileia vastatrix*) | AF339602 | AF339553 | DQ522358 | DQ522406 | DQ522464 |
| *Simplicillium obclavatum* | CBS 311.74 | Air above sugarcane field | AF339567 | AF339517 | EF468798 |  |  |
| *Torrubiella ratticaudata* | ARSEF 1915 | Araneae | DQ522562 | DQ518777 | DQ522360 | DQ522408 | DQ522467 |

Boldface: data generated in this study.
