# Supplementary material for: Species Diversity and Virulence Potential of the Beauveria bassiana Complex and Beauveria scarabaeidicola Complex
Source: Front Microbiol. 2022 Mar 4;13:841604. doi: 10.3389/fmicb.2022.841604 (PMC8934399; doi:10.3389/fmicb.2022.841604)
Supplement: Supplementary file 1 [file Data_Sheet_1.zip › Table S2.docx]

**TABLE S2** Pairwise genetic distance matrix of *Beauveria* species for combined partial *TEF*+*RPB1*+*RPB2*+*Bloc* sequences

| Taxon | 1 | 2 | 3 | 4 | 5 | 6 | 7 | 8 | 9 | 10 | 11 | 12 | 13 | 14 | 15 | 16 | 17 | 18 | 19 | 20 | 21 | 22 | 23 | 24 |
| --- | --- | --- | --- | --- | --- | --- | --- | --- | --- | --- | --- | --- | --- | --- | --- | --- | --- | --- | --- | --- | --- | --- | --- | --- |
| 1 (*B. amorpha*) |  |  |  |  |  |  |  |  |  |  |  |  |  |  |  |  |  |  |  |  |  |  |  |  |
| 2 (*B. araneola*) | 0.033 |  |  |  |  |  |  |  |  |  |  |  |  |  |  |  |  |  |  |  |  |  |  |  |
| 3 (*B. asiatica*) | 0.042 | 0.031 |  |  |  |  |  |  |  |  |  |  |  |  |  |  |  |  |  |  |  |  |  |  |
| 4 (*B. australis*) | 0.036 | 0.015 | 0.031 |  |  |  |  |  |  |  |  |  |  |  |  |  |  |  |  |  |  |  |  |  |
| 5 (*B. bassiana*) | 0.047 | 0.041 | 0.050 | 0.043 |  |  |  |  |  |  |  |  |  |  |  |  |  |  |  |  |  |  |  |  |
| 6 (*B. brongniartii*) | 0.032 | 0.013 | 0.029 | 0.010 | 0.037 |  |  |  |  |  |  |  |  |  |  |  |  |  |  |  |  |  |  |  |
| 7 (*B. caledonica*) | 0.034 | 0.038 | 0.049 | 0.040 | 0.053 | 0.035 |  |  |  |  |  |  |  |  |  |  |  |  |  |  |  |  |  |  |
| 8 (*B. hoplocheli*) | 0.052 | 0.054 | 0.070 | 0.057 | 0.066 | 0.053 | 0.047 |  |  |  |  |  |  |  |  |  |  |  |  |  |  |  |  |  |
| 9 (*B. kipukae*) | 0.045 | 0.038 | 0.050 | 0.037 | 0.037 | 0.033 | 0.044 | 0.060 |  |  |  |  |  |  |  |  |  |  |  |  |  |  |  |  |
| 10 (*B. lii*) | 0.051 | 0.047 | 0.056 | 0.047 | 0.042 | 0.042 | 0.051 | 0.064 | 0.030 |  |  |  |  |  |  |  |  |  |  |  |  |  |  |  |
| 11 (*B. majiangensis*) | 0.042 | 0.027 | 0.010 | 0.029 | 0.047 | 0.026 | 0.048 | 0.067 | 0.047 | 0.053 |  |  |  |  |  |  |  |  |  |  |  |  |  |  |
| 12 (*B. malawiensis*) | 0.078 | 0.076 | 0.092 | 0.076 | 0.081 | 0.076 | 0.065 | 0.069 | 0.076 | 0.083 | 0.088 |  |  |  |  |  |  |  |  |  |  |  |  |  |
| 13 (*B. medogensis*) | 0.035 | 0.010 | 0.034 | 0.019 | 0.044 | 0.016 | 0.038 | 0.054 | 0.039 | 0.050 | 0.029 | 0.076 |  |  |  |  |  |  |  |  |  |  |  |  |
| 14 (*B. peruviensis*) | 0.049 | 0.040 | 0.054 | 0.042 | 0.015 | 0.037 | 0.049 | 0.063 | 0.037 | 0.045 | 0.050 | 0.078 | 0.043 |  |  |  |  |  |  |  |  |  |  |  |
| 15 (*B. polyrhachicola*) | 0.046 | 0.038 | 0.052 | 0.041 | 0.019 | 0.035 | 0.045 | 0.058 | 0.037 | 0.045 | 0.047 | 0.073 | 0.041 | 0.011 |  |  |  |  |  |  |  |  |  |  |
| 16 (*B. pseudobassiana*) | 0.042 | 0.042 | 0.055 | 0.042 | 0.051 | 0.037 | 0.034 | 0.048 | 0.047 | 0.055 | 0.053 | 0.070 | 0.042 | 0.050 | 0.052 |  |  |  |  |  |  |  |  |  |
| 17 (*B. rudraprayagi*) | 0.044 | 0.040 | 0.053 | 0.041 | 0.042 | 0.036 | 0.040 | 0.052 | 0.045 | 0.052 | 0.050 | 0.073 | 0.041 | 0.044 | 0.045 | 0.013 |  |  |  |  |  |  |  |  |
| 18 (*B. scarabaeidicola*) | 0.037 | 0.045 | 0.055 | 0.048 | 0.056 | 0.042 | 0.037 | 0.048 | 0.052 | 0.060 | 0.056 | 0.075 | 0.045 | 0.057 | 0.052 | 0.043 | 0.045 |  |  |  |  |  |  |  |
| 19 (*B. songmingensis*) | 0.039 | 0.048 | 0.059 | 0.050 | 0.060 | 0.046 | 0.040 | 0.052 | 0.053 | 0.062 | 0.060 | 0.079 | 0.048 | 0.058 | 0.054 | 0.047 | 0.049 | 0.013 |  |  |  |  |  |  |
| 20 (*B. staphylinidicola*) | 0.049 | 0.042 | 0.051 | 0.044 | 0.010 | 0.038 | 0.055 | 0.070 | 0.039 | 0.044 | 0.050 | 0.083 | 0.045 | 0.015 | 0.019 | 0.053 | 0.045 | 0.060 | 0.064 |  |  |  |  |  |
| 21 (*B. subscarabaeidicola*) | 0.043 | 0.052 | 0.061 | 0.054 | 0.061 | 0.048 | 0.045 | 0.056 | 0.055 | 0.064 | 0.063 | 0.081 | 0.052 | 0.062 | 0.057 | 0.052 | 0.054 | 0.017 | 0.012 | 0.065 |  |  |  |  |
| 22 (*B. varroae*) | 0.043 | 0.035 | 0.048 | 0.037 | 0.033 | 0.031 | 0.043 | 0.060 | 0.015 | 0.029 | 0.045 | 0.078 | 0.038 | 0.034 | 0.034 | 0.045 | 0.042 | 0.053 | 0.056 | 0.035 | 0.057 |  |  |  |
| 23 (*B. vermiconia*) | 0.038 | 0.041 | 0.052 | 0.043 | 0.051 | 0.037 | 0.019 | 0.054 | 0.046 | 0.055 | 0.049 | 0.071 | 0.041 | 0.048 | 0.044 | 0.041 | 0.043 | 0.039 | 0.043 | 0.053 | 0.047 | 0.045 |  |  |
| 24 (*B. yunnanensis*) | 0.037 | 0.045 | 0.057 | 0.047 | 0.058 | 0.042 | 0.040 | 0.049 | 0.051 | 0.060 | 0.055 | 0.076 | 0.045 | 0.058 | 0.053 | 0.046 | 0.047 | 0.014 | 0.013 | 0.062 | 0.013 | 0.053 | 0.040 |  |
