# Supplementary material for: Species Diversity and Virulence Potential of the Beauveria bassiana Complex and Beauveria scarabaeidicola Complex
Source: Front Microbiol. 2022 Mar 4;13:841604. doi: 10.3389/fmicb.2022.841604 (PMC8934399; doi:10.3389/fmicb.2022.841604)
Supplement: Supplementary file 1 [file Data_Sheet_1.zip › Figure S1.docx]

**
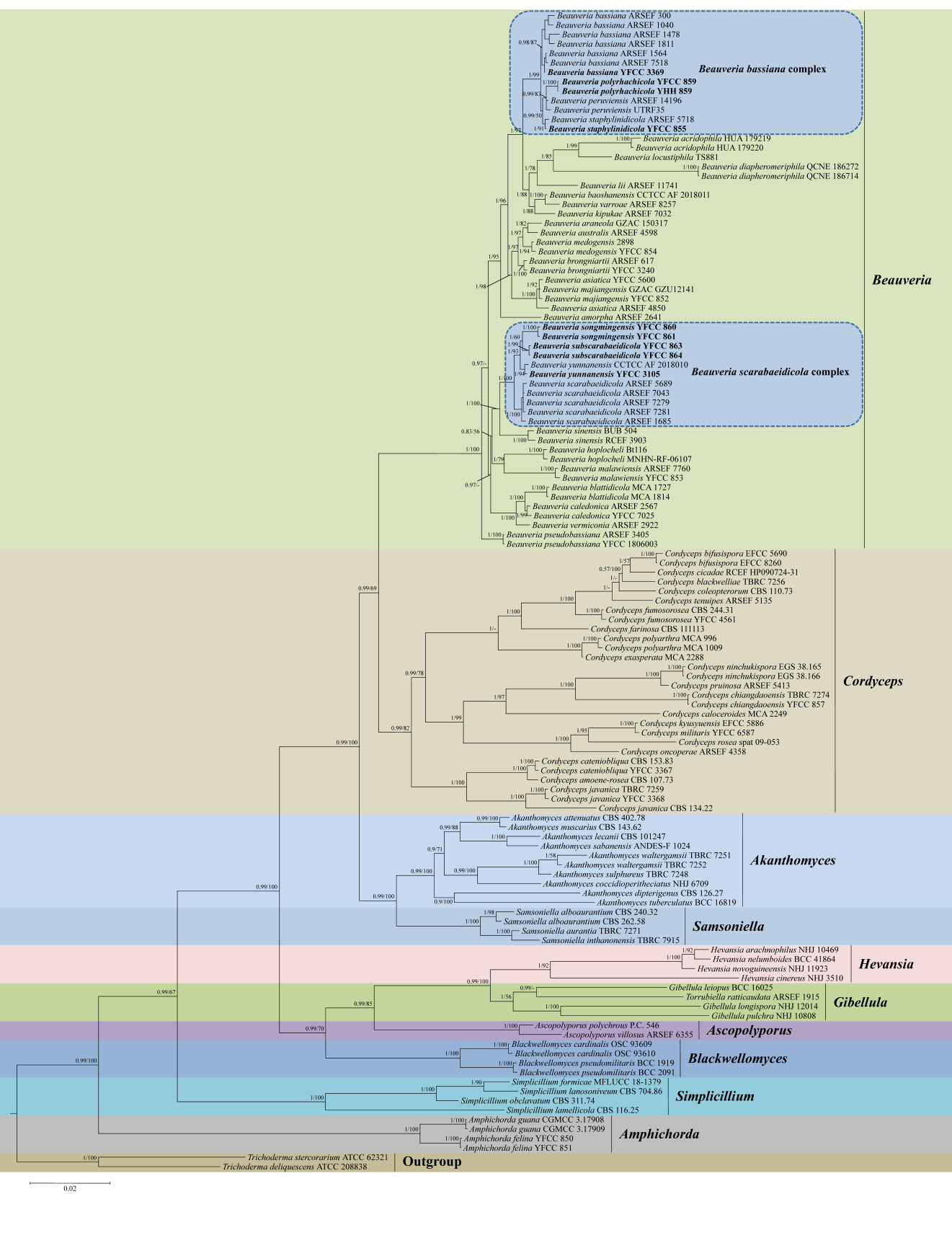
**FIGURE S1 Phylogenetic relationships among the genus *Beauveria* and its allies in Cordycipitaceae based on Bayesian inference (BI) and maximum likelihood (ML) analyses of a five-locus (nr*SSU*, nr*LSU*, *TEF*, *RPB1*, and *RPB2*) dataset. Statistical support values (≥ 0.5/50%) are shown at the nodes for BI posterior probabilities/ML bootstrap support. Isolates in bold type are those analyzed in this study.
